# Amyloid-β probes: Review of structure–activity and brain-kinetics relationships

**DOI:** 10.3762/bjoc.9.116

**Published:** 2013-05-28

**Authors:** Todd J Eckroat, Abdelrahman S Mayhoub, Sylvie Garneau-Tsodikova

**Affiliations:** 1 Department of Pharmaceutical Sciences, University of Kentucky, 789 South Limestone Street, Lexington, KY, 40536-0596, United States; 2 Life Sciences Institute and Department of Medicinal Chemistry, University of Michigan, 210 Washtenaw Ave, Ann Arbor, MI, 48109-2216, United States

**Keywords:** Alzheimer’s disease, in vivo detection, near-infrared fluorescence probes, PET/SPECT imaging, radioactive probes

## Abstract

The number of people suffering from Alzheimer’s disease (AD) is expected to increase dramatically in the coming years, placing a huge burden on society. Current treatments for AD leave much to be desired, and numerous research efforts around the globe are focused on developing improved therapeutics. In addition, current diagnostic tools for AD rely largely on subjective cognitive assessment rather than on identification of pathophysiological changes associated with disease onset and progression. These facts have led to numerous efforts to develop chemical probes to detect pathophysiological hallmarks of AD, such as amyloid-β plaques, for diagnosis and monitoring of therapeutic efficacy. This review provides a survey of chemical probes developed to date for AD with emphasis on synthetic methodologies and structure–activity relationships with regards to affinity for target and brain kinetics. Several probes discussed herein show particularly promising results and will be of immense value moving forward in the fight against AD.

## Introduction

Alzheimer’s disease (AD) is a progressive neurodegenerative disorder of the central nervous system currently affecting ~5.4 million Americans, a number that could increase to 11–16 million by the year 2050. In the United States, AD represents the 6th leading cause of death. Between 2000 and 2008, the number of deaths caused by AD increased by 66%, a dramatic rise, especially when compared to other causes of death, such as heart disease, stroke, prostate and breast cancer, and HIV, which decreased by 3–29% during that time period [1]. As these numbers indicate, AD represents a significant and increasing burden on our population, and efforts towards the development of new and improved diagnostics and therapeutics for this devastating disease are important research endeavors.

Several pathological hallmarks of AD have been identified, and they include decreased cholinergic neurons and acetylcholine (ACh) levels, plaques caused by aggregation of the protein fragment amyloid-β (Aβ), tangles associated with irregular phosphorylation of tau protein, inflammation and increased oxidative stress from reactive oxygen species (ROS), as well as dyshomeostasis and miscompartmentalization of metal ions such as Cu, Fe, and Zn. Observations of these hallmarks have led to several hypotheses in attempts to explain the underlying cause of the disease, which is likely multifactorial. However, the exact cause of AD still remains unknown.

Postmortem histopathological examination of Aβ plaques is currently the only way to firmly confirm AD [2]. In view of the limited accessibility to living brain and other central nervous system (CNS) tissues, AD is currently diagnosed through memory tests and/or based on the patients’ history [2]. Obviously these kinds of diagnostic tools lack absolute sensitivity and accuracy, especially in the early stages of the disease. Therefore, as Aβ plaques precede the onset of dementia and cognitive decline in AD patients, their detection by nuclear imaging techniques such as positron emission tomography (PET) or single-photon emission computed tomography (SPECT) represents the presymptomatic diagnostic tool of choice for AD [3–5].

**Figure 1:**
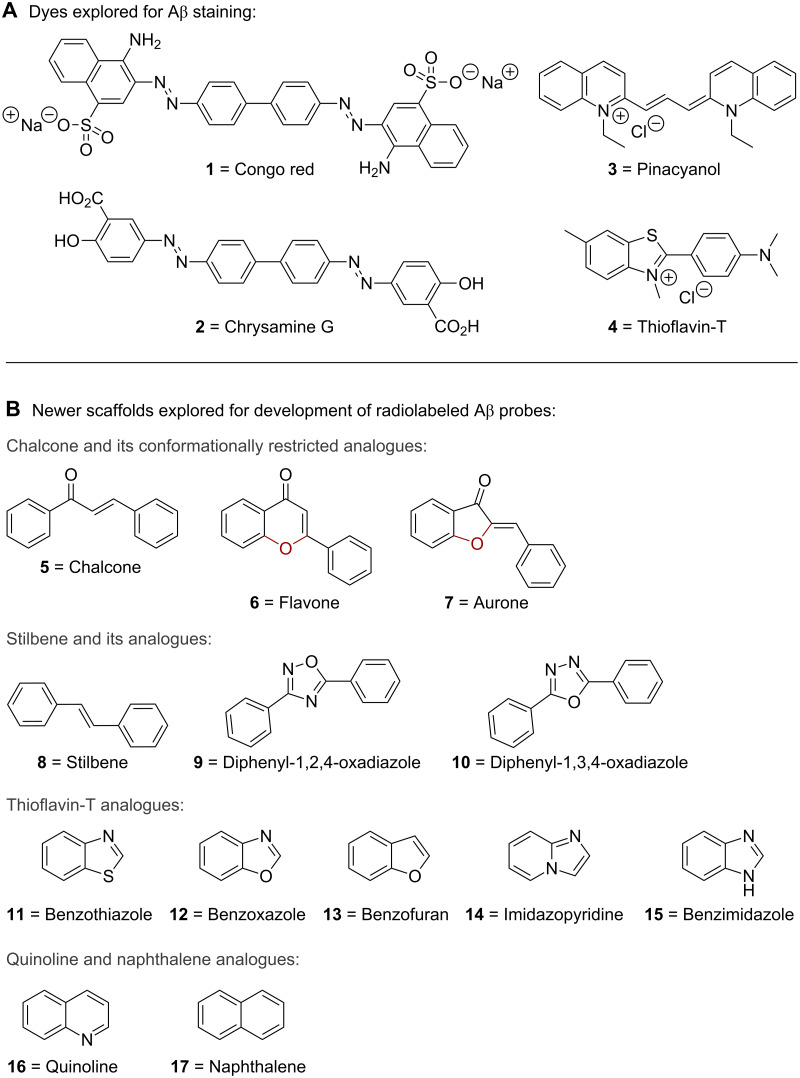
Structures of **A**. dyes originally used to stain Aβ and **B**. newer scaffolds explored for the development of radiolabeled Aβ probes.

The presence of different binding sites in Aβ aggregates led medicinal chemists to investigate and develop a variety of chemical scaffolds as Aβ-imaging tracers [6–9]. To provide a high readable signal-to-background ratio, the ideal Aβ-imaging probes should have certain brain kinetics: a rapid initial brain uptake and a fast washout. Early efforts towards developing Aβ stains focused on dyes such as congo red (**1**), chrysamine G (**2**), pinacyanol (**3**), and thioflavin-T (**4**) (Figure 1A). However, the bulky and ionic natures of these dyes prevented them from crossing the blood brain barrier (BBB), and consequently, no in vivo benefits were obtained from these initial investigations [10–11]. During the past decade, efforts directed at developing probes that display uptake and retention that differ in healthy and AD-affected brains resulted in a variety of radiolabeled molecular probes for in vivo PET/SPECT imaging. The scaffolds from which these newer radiolabeled probes are derived include chalcone (**5**) and its conformationally restricted analogues flavone (**6**) and aurone (**7**); stilbene (**8**) and its analogues diphenyl-1,2,4-oxadiazole (**9**) and diphenyl-1,3,4-oxadiazole (**10**); and thioflavin-T analogues such as benzothiazole (**11**), benzoxazole (**12**), benzofuran (**13**), imidazopyridine (**14**), and benzimidazole (**15**); as well as quinoline (**16**) and naphthalene (**17**) derivatives (Figure 1B). In this review, we provide an overview of these AD radiolabeled early-diagnostic probes according to their scaffolds, with a special emphasis on their synthesis as well as their structure–activity and brain-kinetics relationships. We also provide a brief summary of the latest developments related to the detection of Aβ plaques by near-infrared fluorescence (NIRF) imaging.

## Review

### Radiolabels used in PET/SPECT molecular probes

**Scheme 1:**
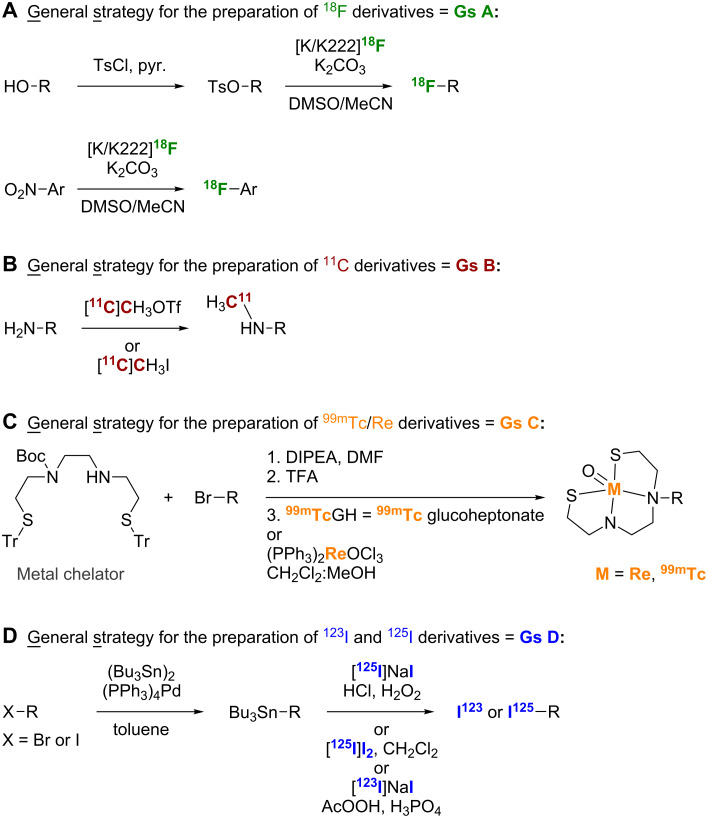
General synthetic strategies (**Gs**) used to introduce **A**. ^18^F, **B**. ^11^C, **C**. ^99m^Tc/Re, and **D**. ^123^I and ^125^I radiolabels into PET probes. Note: **Gs A**–**D** will be used in all subsequent schemes to describe these general synthetic strategies.

Even though they decay rapidly, [^11^C] (*t*_1/2_ = 20 min) and [^18^F] (*t*_1/2_ = 110 min) are the most commonly used radiolabels in PET/SPECT molecular probes for in vivo imaging of Aβ plaques [4]. With a half-life (*t*_1/2_) of 6.01 h compatible with the localization and residence time necessary for imaging, technetium-99m [^99m^Tc] is also a radionuclide of choice that is easily produced by a ^99^Mo/^99m^Tc generator [12]. Iodine isotopes such as [^125^I] are also employed, although much less frequently [13]. The general synthetic methods utilized to introduce radiolabels into PET probes are outlined in Scheme 1. These general strategies will be abbreviated as **Gs A**–**D** in all subsequent schemes in this review.

### Chalcone and its conformationally restricted analogues

#### Chalcone derivatives

**Scheme 2:**
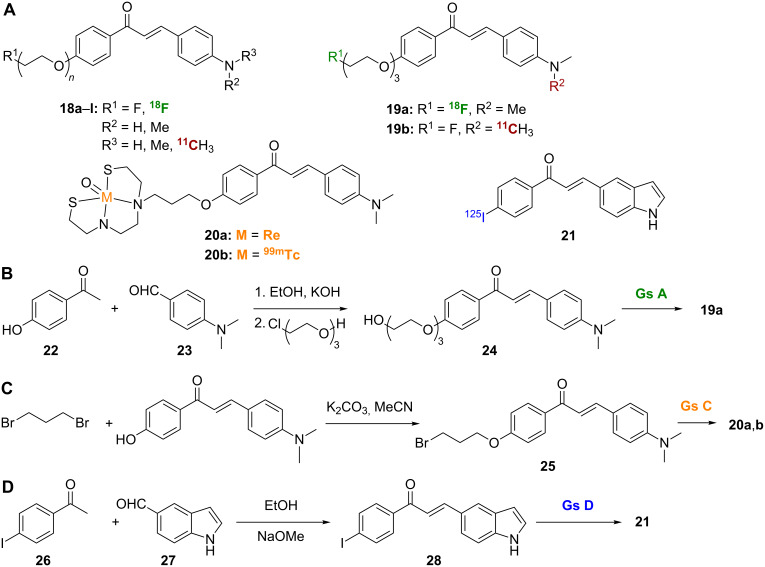
**A**. Structures of radiolabeled chalcone analogues discussed. **B**.–**D**. Synthetic schemes for the preparation of radiolabeled chalcones **19a** and **20a**,**b**, and indolochalcone **21**.

Chalcones and indolochalcones, such as **18a**–**l**, **19a**,**b**, **20a**,**b**, and **21**, have been widely reported as Aβ-imaging tracers (Scheme 2A). Structure–activity-relationship (SAR) studies on fluorinated chalcones **18a**–**l** have shown that, in general, chalcones with tertiary amines in their structures demonstrate good affinity for Aβ plaques in in vitro models (*K*_i_ = 20–50 nM) (Table 1) [14]. Dimethylation of the amino group seems to be crucial for Aβ binding, since analogues with free amino groups or monomethyl amino groups revealed lower affinity [14]. On the other hand, pegylation is not that essential for plaque binding as tertiary amine analogues with different degrees of pegylation (*n* = 0–3) all showed similar affinity. In biodistribution experiments using normal mice, the [^18^F]-labeled chalcone **19a** showed high brain uptake rate and good clearance, whereas the [^11^C]-labeled chalcone **19b** revealed reasonable brain uptake rate, but very fast clearance [14]. The [^18^F]-labeled and [^11^C]-labeled chalcones **19a** and **19b** were synthesized using similar methods, and a representative synthesis of **19a** is shown (Scheme 2B). Aldol condensation between the appropriate acetophenone **22** and benzaldehyde **23** afforded the chalcone backbone, which was subsequently pegylated to give **24** and radiolabeled to give **19a**. Compound **19b** was generated by using *p*-nitrobenzaldehyde instead of the corresponding dimethylamine **23** used in the preparation of **19a** [14]. The resultant nitrochalcone was then reduced by SnCl_2_ in EtOH to yield the free amine, which was monomethylated by controlled addition of an equimolar amount of MeI. The final [^11^C]-labeled compound **19b** was produced by reacting [^11^C]CH_3_OTf with the secondary amine precursor.

**Table 1:** Inhibition constants and biodistribution of radioactivity of fluorinated chalcone derivatives **18a**–**l** and **19a**,**b** (values are from [14]).

Compound	*n*	R^1^	R^2^	R^3^	Aβ_1-42_* K*_i_ (nM)	%ID/g at 2 min	%ID/g at 30 min

**18a**	1	F	Me	Me	45.7 ± 7.1	—	—
[^11^C]**18a**	1	F	Me	**^11^C**H_3_	—	6.01 ± 0.61	2.26 ± 0.41
**18b**	2	F	Me	Me	20.0 ± 2.5	—	—
[^11^C]**18b**	2	F	Me	**^11^C**H_3_	—	4.73 ± 0.47	1.00 ± 0.19
**18c**	3	F	Me	Me	38.9 ± 4.2	—	—
**19a**	3	**^18^F**	Me	Me	—	3.48 ± 0.47	1.07 ± 0.17
**19b**	3	F	Me	**^11^C**H_3_	—	4.31 ± 0.33	0.35 ± 0.03
**18d**	1	F	H	H	678.9 ± 21.7	—	—
**18e**	2	F	H	H	1048.0 ± 114.3	—	—
**18f**	3	F	H	H	790.0 ± 132.1	—	—
**18g**	1	F	H	Me	197.1 ± 58.8	—	—
**18h**	2	F	H	Me	216.4 ± 13.8	—	—
**18i**	3	F	H	Me	470.9 ± 100.4	—	—
**18j**	0	F	Me	Me	49.8 ± 6.2	—	—
[^11^C]**18j**	0	F	Me	**^11^C**H_3_	—	3.68 ± 0.35	1.04 ± 0.20
**18k**	0	F	H	H	663.0 ± 88.3	—	—
**18l**	0	F	H	Me	234.2 ± 44.0	—	—

[Re]- and [^99m^Tc]-labeled chalcone analogues **20a** and **20b** were also studied (Scheme 2A) [12]. The [Re]-labeled analogue **20a** displayed higher affinity for Aβ plaque than did the corresponding [^99m^Tc]-derived compound **20b**. However, **20b** showed better brain pharmacokinetics than **20a**, as indicated by its high brain-uptake rate (1.48% ID/g) and rapid wash out from the CNS (0.17% ID/g at 60 min). Compounds **20a** and **20b** were synthesized by reacting a Boc-protected metal chelator (Scheme 1C) with 4-*O*-(bromopropyl)hydroxychalcone **25** (Scheme 2C). After removal of the Boc protecting group, the final [Re]- and [^99m^Tc]-labeled chalcones **20a** and **20b** were obtained by treatment with (PPh_3_)_2_ReOCl_3_ and ^99m^TcGH, respectively [12].

Finally, the radioiodinated indolochalcone **21**, among a series of other derivatives, was prepared through condensation of 4-iodoacetophenone (**26**) and indole-5-carboxaldehyde (**27**) to give **28**, which was radiolabeled to give the target compound (Scheme 2D) [15]. The indolochalcone **21** showed good binding affinity for Aβ_1-42_ aggregates with a *K*_i_ < 10 nM. Replacement of the iodo substituent with a chloro, bromo, methoxy, or dimethylamino substituent all gave similar results, but replacement with a fluoro, hydroxy, amino, or methylamino substituent all reduced affinity to varying degrees. Autoradiography in sections of brain tissue from an AD animal model showed that **21** specifically labeled Aβ plaques, but its efficacy was hampered by low in vivo uptake into the brain (0.41% ID/g at 2 min) [15].

#### Conformationally restricted chalcones: flavones and aurones

**Scheme 3:**
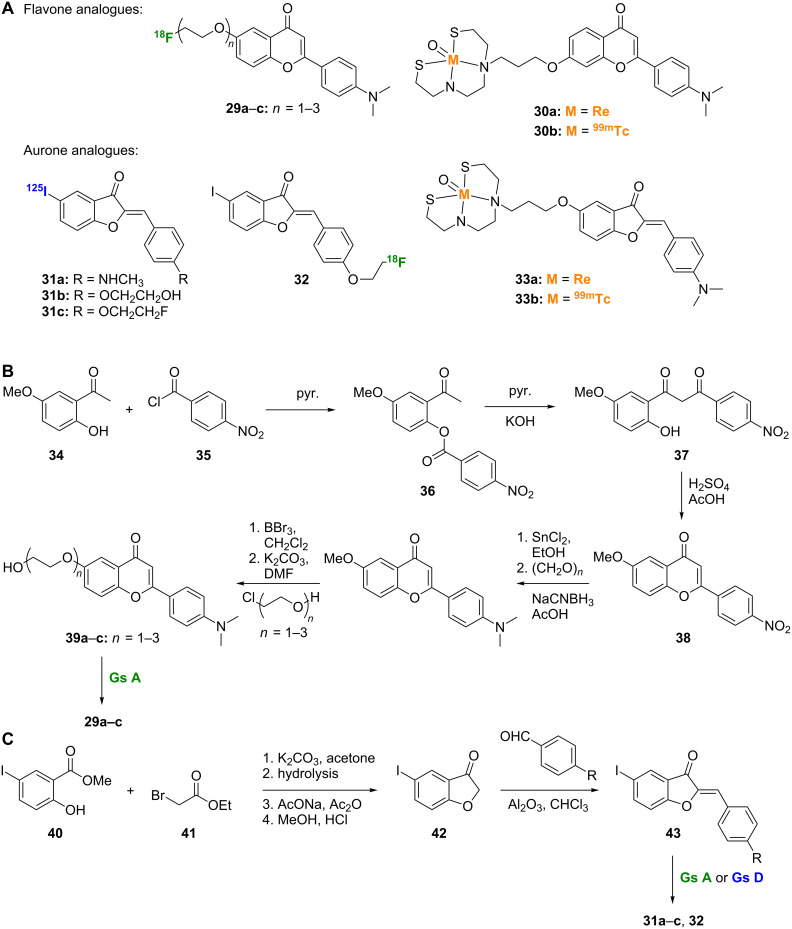
**A**. Structures of the radiolabeled flavone and aurone analogues discussed. **B**. Synthetic scheme for the preparation of [^18^F]-labeled flavone derivatives. **C**. Synthetic scheme for the preparation of the aurone scaffold and its [^18^F]- and [^125^I]-labeled analogues.

Flavones and aurones, such as **29a**–**c**, **30a**,**b**, **31a**–**c**, **32**, and **33a**,**b** (Scheme 3A), can be classified as conformationally restricted chalcone derivatives as their basic structures result from insertion of an oxygen atom between the double bond and the phenyl ring attached to the carbonyl group of the chalcone scaffold (Figure 1B, with oxygen atoms depicted in red). The affinity of flavonoids towards Aβ aggregates was first established by using fluorescence staining in brain sections of Tg2576 transgenic mice [10]. The absence of spots in wild-type mouse brain sections indicated the specificity of flavonoids towards Aβ aggregates in AD mouse models. The [^18^F]-labeled pegylated flavones **29a**–**c** showed high affinity towards Aβ aggregates with *K*_i_ values ranging between 5.3 nM for **29a** and 19.3 nM for **29c** (Scheme 3A) [16]. SAR studies suggest that, as with chalcones, the tertiary amine in these flavones was important for binding and tracing Aβ aggregates in mouse models, as they consistently outperformed secondary and primary amine analogues [16]. Also as with chalcones, the degree of pegylation had only minor effects on binding properties. Compounds **29a**–**c** showed uptake rates indicative of high to sufficient levels for brain imaging (2.89–4.17% ID/g at 2 min) and moderate clearance rates [16]. The flavone backbone of **29a**–**c** was built by acylating 2-hydroxy-5-methoxyacetophenone (**34**) with 4-nitrobenzoyl chloride (**35**) and subjecting the resulting 2-acyloxyacetophenone (**36**) to Baker–Venkataraman rearrangement [17] to afford the 1,3-diarylpropane-1,3-dione **37**, which was dehydrated with sulfuric acid to give **38**. Subsequent nitro reduction, reductive methylation, methyl ether cleavage, and pegylation gave the nonlabeled precursors **39a**–**c**. The [^18^F]-label was introduced by using the standard [K/K222]^18^F in DMSO and acetonitrile reaction conditions (Scheme 3B) [16]. The [Re]- and [^99m^Tc]-labeled flavone complexes **30a** and **30b** were also prepared by using the procedure described for the synthesis of the [Re]- and [^99m^Tc]-labeled chalcones **20a** and **20b** (Scheme 2). The [^99m^Tc]-labeled flavone complex **30a** displayed high Aβ plaque affinity but limited brain uptake [18].

Aurone derivatives have been investigated for their Aβ plaque binding affinity [19]. The [^125^I]-labeled methylamine aurone **31a** presented great binding affinity to Aβ aggregates (*K*_i_ = 1.2 nM), better than all reported flavones to date. It also showed rapid brain uptake rate (3.17% ID/g at 2 min) and rapid clearance (0.24% ID/g at 60 min) [19]. The effect of the tertiary amine in this aurone scaffold was less pronounced than that seen with chalcones or flavones. The dimethylamine analogue of **31a** had approximately six times weaker binding affinity, while the free amine analogue showed only two times weaker affinity. To further enhance the Aβ plaque traceability of **31a**, its methylamine moiety was replaced with ethylene oxide to provide compound **31b**, which exhibited a *K*_i_ value of 1.05 nM in an in vitro binding assay [13]. The brain kinetics of **31b** (brain uptake = 4.51% ID/g at 2 min and washout = 0.09% ID/g at 60 min) were found to be slightly better than those of **31a** [13]. Addition of 2 or 3 ethylene oxide units or replacement with a hydroxy or methoxy group did not significantly improve the plaque binding affinity and modestly affected the brain kinetics. Replacement of the terminal hydroxy group of **31b** with a fluorine atom negatively affected the brain uptake property of compound **31c** (2.34% ID/g at 2 min) when compared to **31b** [20]. However, it did not affect the washout character of the compound. These results were confirmed by preparation and analysis of the [^18^F]-labeled compound **32** [20]. In general, the aurone derivatives **31a**–**c** and **32** were built from the reaction of methyl 5-iodosalicilate (**40**) with ethyl bromoacetate (**41**) followed by ester hydrolysis and cyclization to afford 5-iodo-3-coumaranone (**42**), which, after condensation with the proper benzaldehydes, gave the aurone scaffold **43**, which could be radiolabeled (Scheme 3C). As for the chalcone and flavone derivatives, [Re]- and [^99m^Tc]-labeled aurone complexes **33a** and **33b** were also prepared [18]. The high affinity for Aβ aggregates observed with the [^99m^Tc]-labeled aurone **33b** was hampered by its weak brain penetration, which made it unsuitable for in vivo application [18].

### Stilbene and its analogues

#### Stilbene derivatives

**Scheme 4:**
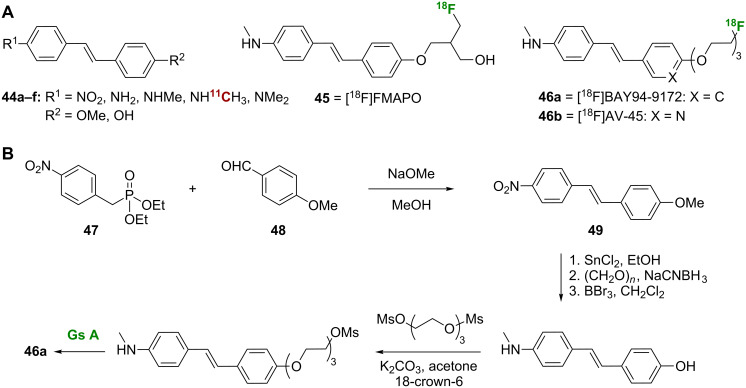
**A**. Structures of the radiolabeled stilbene analogues discussed. **B**. Synthetic scheme for the preparation of [^18^F]BAY94-9172 (**46a**).

The SARs of stilbene analogues, such as **44a**–**f**, **45**, and **46a**,**b** (Scheme 4A), as Aβ plaque tracers have been thoroughly investigated. In general, it was found that an electron-donating group at each end of the stilbene derivative is essential for Aβ plaque binding affinity [21]. Analysis of stilbenes **44a–f** shows that a monomethylated or dimethylated amine at one end of the stilbene core leads to strong binding affinity for Aβ_1-40_ aggregates, while a free amine or nitro group reduces affinity. The opposite end of the stilbene core can be substituted with a hydroxy or methoxy substituent with little effect on binding affinity (Table 2). Derivative **44d**, which showed good affinity towards Aβ aggregates in vitro (*K*_i_ = 6.0 ± 1.5 nM), has been radiolabeled to give *N*-[^11^C]methylamino-4'-hydroxystilbene ([^11^C]**44d**), and this compound shows excellent labeling of Aβ plaques in TgCRDN8 mouse brain sections by in vitro autoradiography [22].

**Table 2:** Inhibition constants and biodistribution of radioactivity of stilbene derivatives **44a**–**f** and [^11^C]**44d** (values are from [22]).

Compound	R^1^	R^2^	Aβ_1-40_* K*_i_ (nM)	%ID/g at 2 min	%ID/g at 60 min

**44a**	NO_2_	OMe	151 ± 30	—	—
**44b**	NH_2_	OMe	36 ± 5	—	—
**44c**	NHMe	OMe	1.2 ± 0.5	—	—
**44d**	NHMe	OH	6.0 ± 1.5	—	—
[^11^C]**44d**	NH**^11^C**H_3_	OH	—	1.15 ± 0.08	0.30 ± 0.03
**44e**	NMe_2_	OMe	1.3 ± 0.4	—	—
**44f**	NMe_2_	OH	2.2 ± 0.6	—	—

[^18^F]-Labeled stilbene derivatives have enhanced brain kinetics rendering them appropriate for clinical use [23–25]. In order to control the lipophilicity and keep the partition coefficient (log *P*) value between 1 and 3, which reduces brain nonspecific binding and improves signal-to-noise ratio, additional hydroxy or ethylene oxide unit(s) were added [21]. An early fluorinated stilbene was [^18^F]FMAPO (**45**), which demonstrated high binding affinity for Aβ aggregates (*K*_i_ = 5.0 ± 1.2 nM) in assays using human AD brain homogenates [26]. Even though addition of the fluoroalkyl side chain moiety had little effect on the binding affinity and the clearance rate, it improved brain kinetics significantly (from 1.15% ID/g at 2 min for [^11^C]**44d** [22] to 9.75% ID/g at 2 min for **45** [26]). Florbetaben ([^18^F]BAY94-9172, **46a**), another member of the stilbene class, showed strong binding affinity for human AD brain homogenates (*K*_i_ = 6.7 ± 0.3 nM) and promising pharmacokinetics [21], and this compound has progressed to clinical trials. Compound **46a** was tested clinically on 15 AD patients and a similar number of healthy elderly volunteers [4]. Interestingly, all AD patients showed widespread neocortical binding of **46a**, which was quantified by using the standardized uptake value ratio (SUVR) technique [4]. This observation was further supported by another study using a wider sample population where AD patients demonstrated significantly higher SUVRs when compared to healthy patients or patients with other neural diseases such as Parkinson’s disease, mild cognitive impairment, frontotemporal lobar degeneration, dementia with Lewy bodies, and vascular dementia [27]. More recent phase 2/3 clinical trials collectively showed that compound **46a** displays a high degree of sensitivity and selectivity in discriminating between patients with probable AD and age-matched healthy controls [28].

A pyridine analogue of **46a**, florbetapir ([^18^F]AV-45, **46b**) was also prepared using a tosylate precursor with Sumitomo modules for radiosynthesis [23,29]. Compound **46b** displayed strong affinity for Aβ peptides in AD brain homogenates (*K*_i_ = 2.87 ± 0.17 nM), excellent pharmacokinetics [30], and an acceptable safety profile that paved the way to its clinical application in brain imaging [31]. A number of **46b**/PET studies have been conducted [23,32–39]. Using **46b** as an imaging probe, PET indicated that the drug accumulates explicitly in Aβ-deposition-rich cortical regions in AD patients with minimal accumulation observed in healthy volunteers [40].

In general, the stilbene nucleus was built using the Wadsworth–Emmons reaction, and a representative synthesis of stilbene **46a** is shown (Scheme 4B). Initial Wadsworth–Emmons reaction between diethyl (4-nitrobenzyl)phosphonate (**47**) and 4-methoxybenzaldehyde (**48**) constructed the stilbene core **49**. The target compound **46** was formed from a straightforward sequence of nitro reduction, reductive methylation, methyl ether cleavage, pegylation and radiolabeling. Several synthetic procedures have been described for the preparation of **46a** and its precursors in an effort to optimize yield [21,41–42]. The best reported yield and purity was obtained by mixing the mesylate precursor with the fluorinating agent in a modified PET-MF-2V-IT-1 synthesizer and by purifying using plus C18 Sep-Pak cartridges [41]. In the preparation of [^11^C]**44d**, the [^11^C]-methylation of 4-amino-4'-hydroxystilbene was carried out using the “LOOP” method, in which trapping and reaction of [^11^C]CH_3_OTf with the appropriate stilbene analogue takes place inside an HPLC sample loop [43].

#### Diphenyl-1,2,4- and diphenyl-1,3,4-oxadiazoles

**Scheme 5:**
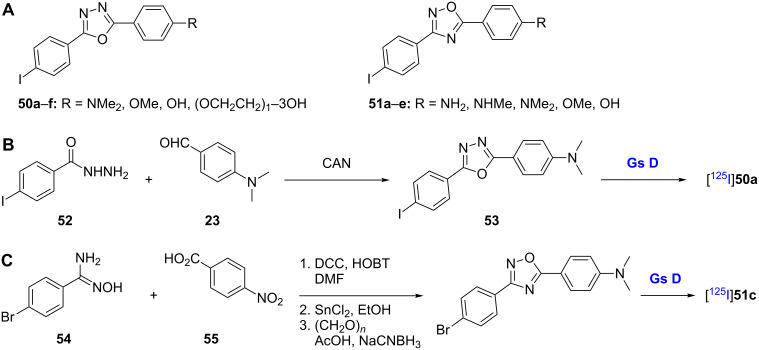
**A**. Structures of the diphenyl-1,3,4- and diphenyl-1,2,4-oxadiazoles discussed. **B**.,**C**. Synthetic schemes for the preparation of [^125^I]**50a** and [^125^I]**51c**, respectively.

The replacement of the stilbene ethylene linker with different heterocycles is a common strategy in medicinal chemistry used to improve the pharmacokinetics and/or pharmacodynamics of stilbenes (Scheme 5A) [44–46]. In the case of Aβ probes, a series of 2,5-diphenyl-1,3,4-oxadiazoles **50a**–**f** and 3,5-diphenyl-1,2,4-oxadiazoles **51a**–**e** have been studied in this respect (Table 3 and Table 4). Among the 2,5-diphenyl-1,3,4-oxadiazoles, the dimethylamine analogue **50a** (*K*_i_ = 20.1 ± 2.5 nM) and methoxy analogue **50b** (*K*_i_ = 46.1 ± 12.6 nM) showed the best affinities towards Aβ aggregates, and radiolabeling has been performed for both of these compounds. In biodistribution studies, the dimethylamine analogue [^125^I]**50a** showed good brain uptake and washout rates. Although methoxy analogue [^125^I]**50b** showed poorer brain uptake, its washout rate was increased compared to its dimethylamine counterpart [47]. Interestingly, changing the heteroatom order in the central ring from 1,3,4 (**50a**–**f**) to 1,2,4 (**51a**–**e**) has great effects on both the physical characteristics and pharmacokinetics of the compounds. The 3,5-diphenyl-1,2,4-oxadiazole analogue **51c** was more lipophilic than its 1,3,4 counterpart **50a** (log *P* = 3.22 for **51c** and 2.43 for **50a**) [47]. In general, even though 3,5-diphenyl-1,2,4-oxadiazoles **51a**–**e** show excellent affinity for Aβ aggregates in in vitro binding experiments (*K*_i_ = 4.3–47.1 nM), they show poorer brain uptake rates (1.07–2.06% ID/g at 2 min) and slower washout rates (3.29–2.01% ID/g at 60 min) than their 1,3,4 counterparts [48]. These findings, together with the close structural similarities between compounds **50a**–**f** and **51a**–**e**, highlight the importance of lipophilicity as a factor in controlling brain kinetics [47].

**Table 3:** Inhibition constants and biodistribution of radioactivity of the 2,5-diphenyl-1,3,4-oxadiazole derivatives **50a**–**f** (values are from [47]).

Compound	R	Aβ_1-42_* K*_i_ (nM)	%ID/g at 10 min	%ID/g at 60 min

**50a**	NMe_2_	20.1 ± 2.5	—	—
[^125^I]**50a**	NMe_2_	—	5.93 ± 0.76	1.78 ± 0.41
**50b**	OMe	46.1 ± 12.6	—	—
[^125^I]**50b**	OMe	—	2.74 ± 0.37	0.36 ± 0.13
**50c**	OH	229.6 ± 47.3	—	—
**50d**	OCH_2_CH_2_OH	282.2 ± 61.4	—	—
**50e**	(OCH_2_CH_2_)_2_OH	348.6 ± 51.7	—	—
**50f**	(OCH_2_CH_2_)_3_OH	257.7 ± 34.8	—	—

**Table 4:** Inhibition constants and biodistribution of radioactivity of 3,5-diphenyl-1,2,4-oxadiazole derivatives **51a**–**e** (values are from [48]).

Compound	R	Aβ_1-42_* K*_i_ (nM)	%ID/g at 2 min	%ID/g at 60 min

**51a**	NH_2_	14.2 ± 1.4	—	—
[^125^I]**51a**	NH_2_	—	1.61 ± 0.23	3.29 ± 0.58
**51b**	NHMe	14.3 ± 3.6	—	—
[^125^I]**51b**	NHMe	—	1.44 ± 0.12	2.70 ± 0.33
**51c**	NMe_2_	15.4 ± 1.4	—	—
[^125^I]**51c**	NMe_2_	—	1.07 ± 0.23	2.32 ± 0.64
**51d**	OMe	4.3 ± 2.1	—	—
[^125^I]**51d**	OMe	—	2.06 ± 0.45	2.01 ± 0.33
**51e**	OH	47.1 ± 4.1	—	—

Representative syntheses of radioiodinated oxadiazoles **50a** and **51c** are shown (Scheme 5B and C). The 1,3,4-oxadiazole core of [^125^I]**50a** was obtained from the reaction between 4-iodobenzhydrazide (**52**) and 4-dimethylaminobenzaldehyde (**23**) in the presence of ceric ammonium nitrate (CAN) followed by subsequent radioiodination of compound **53** (Scheme 5B) [47]. The 1,2,4-oxadiazole core of [^125^I]**51c** was obtained by DCC/HOBt-mediated condensation of 4-bromobenzamidoxime (**54**) and *p*-nitrobenzoic acid (**55**). Subsequent nitro reduction, reductive methylation, and radioiodination gave [^125^I]**51c** (Scheme 5C) [48].

### Thioflavin-T analogues

#### Benzothiazoles

**Figure 2:**
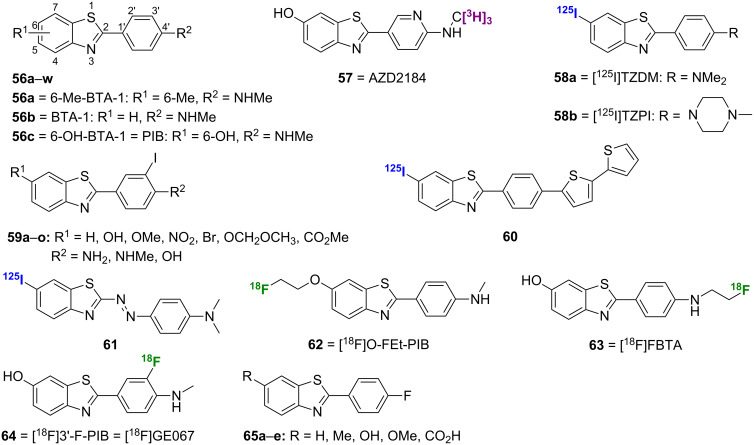
Structures of the radiolabeled benzothiazole analogues discussed.

Of all the amyloid imaging classes, the benzothiazoles, such as **56a**–**w**, **57**, **58a**,**b**, and **59**–**65** (Figure 2), may well be one of the most prolific and well-studied. The amyloid imaging dye thioflavin-T (**4**, Figure 1) served as the inspiration for this class of radiotracers in which the ionic charge was removed to increase lipophilicity and to enhance in vivo BBB permeability. Overall, this class of compounds shows high affinity for Aβ aggregates with promising in vivo pharmacokinetics.

**Scheme 6:**
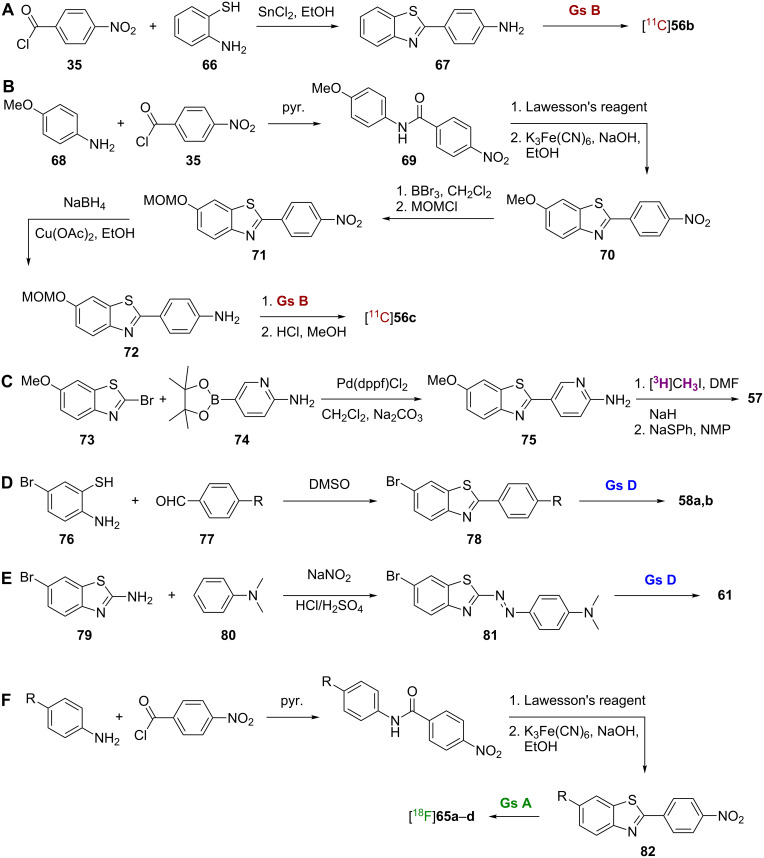
**A**.–**F**. Synthetic schemes for the preparation of [^11^C]**56b**, [^11^C]**56c**, **57**, **58a,b**, **61**, and [^18^F]**65a–d**.

One of the earliest radiolabeled benzothiazoles, [^11^C]6-Me-BTA-1 ([^11^C]**56a**; note: BTA = 2-(4'-methylaminophenyl)benzothiazole, Figure 2), was prepared by methylation of 4-(6-methyl-2-benzothiazolyl)aniline using [^11^C]methyl iodide [49]. Compared to **4**, **56a** showed greatly increased lipophilicity and improved binding affinity for Aβ_1-40_ (*K*_i_ = 890 nM for **4** and *K*_i_ = 20.2 for **56a**). In postmortem AD brain sections, [^11^C]**56a** was able to stain both Aβ plaques and neurofibrillary tangles (NFTs), while pharmacokinetic studies in normal mice showed high brain uptake (7.61% ID/g at 2 min) and good washout (2.76% ID/g at 30 min). Additional modification of this scaffold by removal of the 6-Me group gave [^11^C]BTA-1 ([^11^C]**56b**) [50]. Compound **56b** was prepared by coupling of *p*-nitrobenzoyl chloride (**35**) and 2-aminothiophenol (**66**) followed by nitro reduction to **67** and methylation using [^11^C]methyl iodide (Scheme 6A). While showing a near equal binding affinity for Aβ, the decreased lipophilicity of [^11^C]**56b** to the ideal level led to improved pharmacokinetics over [^11^C]**56a** as evidenced by improved uptake and washout rates in normal mice (12.9% ID/g at 2 min and 1.7% ID/g at 30 min). Compound [^11^C]**56b** showed in vivo specificity for Aβ in the brains of PS1/APP transgenic mice, and it was subsequently shown to bind specifically to amyloid deposits in human AD brain homogenates [51].

The addition of a hydroxy group at the 6-position of [^11^C]**56b** gave [^11^C]6-OH-BTA-1 ([^11^C]**56c**) [52]. Compound [^11^C]**56c** was synthesized by first coupling *p*-anisidine (**68**) with *p*-nitrobenzoyl chloride (**35**) to give the amide **69**, which was subsequently converted to the thioamide by using Lawesson’s reagent and cyclized to form the benzothiazole core **70** (Scheme 6B). Demethylation with BBr_3_ and protection of the resulting hydroxy moiety as the methoxymethyl (MOM) ether gave **71**. Reduction of the nitro group to **72**, methylation using [^11^C]methyl iodide, and cleavage of the MOM ether gave [^11^C]**56c**. Compound **56c** showed high affinity for Aβ_1-40_ (*K*_i_ = 4.3 nM) (Table 5). This synthesis has since been refined to improve radiochemical yields and eliminate the need for a protecting group by use of [^11^C]CH_3_OTf as the methylating agent [53].

**Table 5:** Inhibition constants and biodistribution of radioactivity of 6-substituted 2-arylbenzothiazole derivatives **56c**–**t** (values are from [52]).

Compound	R^1^	R^2^	Aβ_1-40_* K*_i_ (nM)	(%ID-kg)/g at 2 min	(%ID-kg)/g at 30 min

**56c**	6-OH	NHMe	4.3	—	—
[^11^C]**56c**	6-OH	NH**^11^C**H_3_	—	0.21	0.018
**56d**	6-OH	NH_2_	46	—	—
**56e**	6-OH	NMe_2_	4.4	—	—
[^11^C]**56e**	6-OH	NMe**^11^C**H_3_	—	0.32	0.10
**56f**	6-H	NHMe	11	—	—
[^11^C]**56f**	6-H	NH**^11^C**H_3_	—	0.43	0.057
**56g**	6-H	NH_2_	37	—	—
**56h**	6-H	NMe_2_	4.0	—	—
[^11^C]**56h**	6-H	NMe**^11^C**H_3_	—	0.19	0.078
**56i**	6-Me	NHMe	10	—	—
[^11^C]**56i**	6-Me	NH**^11^C**H_3_	—	0.22	0.083
**56j**	6-Me	NH_2_	9.5	—	—
**56k**	6-Me	NMe_2_	64	—	—
[^11^C]**56k**	6-Me	NMe**^11^C**H_3_	—	0.078	0.15
**56l**	6-OMe	NHMe	4.9	—	—
[^11^C]**56l**	6-OMe	NH**^11^C**H_3_	—	0.33	0.10
**56m**	6-OMe	NH_2_	7.0	—	—
[^11^C]**56m**	6-O**^11^C**H_3_	NH_2_	—	0.32	0.084
**56n**	6-OMe	NMe_2_	1.9	—	—
[^11^C]**56n**	6-OMe	NMe**^11^C**H_3_	—	0.16	0.14
**56o**	6-CN	NHMe	8.6	—	—
[^11^C]**56o**	6-CN	NH**^11^C**H_3_	—	0.32	0.063
**56p**	6-CN	NH_2_	64	—	—
**56q**	6-CN	NMe_2_	11	—	—
[^11^C]**56q**	6-CN	NMe**^11^C**H_3_	—	0.24	0.097
**56r**	6-Br	NHMe	1.7	—	—
[^11^C]**56r**	6-Br	NH**^11^C**H_3_	—	0.12	0.12
**56s**	6-Br	NH_2_	7.2	—	—
**56t**	6-Br	NMe_2_	2.9	—	—
[^11^C]**56t**	6-Br	NMe**^11^C**H_3_	—	0.054	0.11

The 6-OH group of **56c** made it less lipophilic than both **56a** and **56b** and likely contributed to its moderate brain entry (0.21% ID-kg/g at 2 min) but good clearance (0.018% ID-kg/g at 30 min) in normal mice. Interesting SAR findings on this scaffold from comparison of **56c**–**t** (Table 5) included that the more lipophilic secondary and tertiary amines at the 4'-position were more potent (*K*_i_) than primary amines. Also, in general, substitution at the 6-position seemed to have only a small effect in terms of *K*_i_ as 6-OH, -OCH_3_, -CN, and -Br gave similar results. However, substitution at the 6-position had a larger effect on pharmacokinetics in the brain, as 6-OH clearly gave the best results [52]. As one of the most successful radiolabeled Aβ imaging probes to date, [^11^C]**56c** has subsequently been named Pittsburgh Compound B (PIB).

Additional studies of [^11^C]**56c** in humans have been promising and suggest that PET imaging with this compound can provide quantitative information on amyloid deposits in living patients. In postmortem tissue, [^11^C]**56c** exhibited specific binding to the amyloid-laden frontal cortex of the AD brain, but little binding to the frontal cortex of the cognitively normal age-matched control brain. Compound [^11^C]**56c** also displayed a rapid entry and clearance in the brain of healthy controls, but a marked retention in AD patients in areas of the brain known to contain large amyloid deposits [54]. Additional data suggested that [^11^C]**56c** was suitable for early detection of pathological changes in AD patients before a significant loss of cognitive function is apparent [55].

The impact of changing the position of the hydroxy group of **56c** was investigated by synthesizing the 4-OH, 5-OH, and 7-OH analogues **56u**–**w** using methods similar to those described above [56]. The *K*_i_ values for these analogues in human AD brain homogenates were between 11–19 nM, indicating slightly reduced affinity compared to **56c** (*K*_i_ = 2.8 nM) (Table 6). However, each radiolabled analogue was able to stain plaques in sections from transgenic AD mouse brain and human AD brain. The 5-OH analogue [^11^C]**56v** showed the best pharmacokinetic profile in normal mice with high brain uptake and a washout rate, that was 8 times faster than that of [^11^C]**56c**. Interestingly, it was noted that the 4-OH analogue **56u** could form an intramolecular hydrogen bond (i.e. an extra pseudo ring), which could act to increase the lipophilicity of the compound and lead to nonspecific binding and residual background activity in the brain.

**Table 6:** Inhibition constants and biodistribution of radioactivity of hydroxy-substituted 2-arylbenzothiazole derivatives **56c**,**u**–**w** (values are from [56]).

Compound	R^1^	R^2^	human AD brain homogenates *K*_i_ (nM)	%ID/g at 2 min	%ID/g at 60 min

**56c**	6-OH	NHMe	2.8 ± 0.5	—	—
[^11^C]**56c**	6-OH	NH**^11^C**H_3_	—	3.6 ± 1.4	0.6 ± 0.2
**56u**	4-OH	NHMe	18.8 ± 3.8	—	—
[^11^C]**56u**	4-OH	NH**^11^C**H_3_	—	3.8 ± 0.9	0.3 ± 0.3
**56v**	5-OH	NHMe	11.5 ± 3	—	—
[^11^C]**56v**	5-OH	NH**^11^C**H_3_	—	4.3 ± 0.45	0.09 ± 0.02
**56w**	7-OH	NHMe	11.2 ± 5	—	—
[^11^C]**56w**	7-OH	NH**^11^C**H_3_	—	2.6 ± 0.76	0.16 ± 0.03

A [^3^H]-labeled analogue of **56c**, AZD2184 (**57**), was also synthesized to give a higher signal-to-background ratio by virtue of its decreased lipophilicity [57]. This compound was prepared through palladium catalyzed Suzuki coupling of the starting halide **73** and boronic acid **74** followed by *N*-methylation of **75** with [^3^H]methyl iodide and *O*-demethylation with sodium thiophenoxide (Scheme 6C). Compound **57** showed high affinity for Aβ_1-40_ fibrils in vitro (*K*_d_ = 8.4 nM) and lower background binding levels than **56c**. While **57** was able to label amyloid deposits in APP/PS1 mice, its brain penetration was not as high as that of [^11^C]**56c**.

Besides [^11^C], other radiolabels have been investigated for benzothiazole imaging agents. Two of the earliest [^125^I]-labeled imaging agents reported were [^125^I]TZDM (**58a**) and [^125^I]TZPI (**58b**) [58]. The synthesis of these agents was achieved in two steps by condensation of 5-bromo-2-aminobenzenethiol (**76**) and the appropriate benzaldehyde **77** followed by radiolabeling of **78** (Scheme 6D). Both **58a** and **58b** showed high affinity for Aβ_1-40_ and Aβ_1-42_ aggregates with *K*_d_ values ≤0.15 nM in all cases. However, pharmacokinetics for these agents were less than ideal as both showed long retention in the brains of normal mice, which is indicative of nonspecific binding.

A series of iodinated benzothiazoles **59a**–**o** was synthesized using methods similar to those described above and SAR studies were performed (Table 7) [59]. Among the interesting findings was that the introduction of 3'-iodo increased lipophilicity and binding to Aβ_1-40_ when R^2^ = NHMe. However, the opposite effect on binding was observed when R^2^ = OH. Among the [^125^I]-labeled derivatives, more polar compounds exhibited better clearance and less nonspecific binding in the brains of normal mice, a typical result for brain imaging probes. One of the most promising compounds identified in this study was [^125^I]**59d**.

**Table 7:** Inhibition constants and biodistribution of radioactivity of iodinated 2-arylbenzothiazole derivatives **59a**–**o** (values are from [59]).

Compound	R^1^	R^2^	Aβ_1-40_* K*_i_ (nM)	%ID/g at 2 min	%ID/g at 30 min

**59a**	H	NH_2_	8.32	—	—
[^125^I]**59a**	H	NH_2_	—	9.08	3.4
**59b**	H	NHMe	4.94	—	—
[^125^I]**59b**	H	NHMe	—	4.40	2.68
**59c**	H	OH	19.1	—	—
**59d**	OH	NH_2_	11.1	—	—
[^125^I]**59d**	OH	NH_2_	—	5.64	0.36
**59e**	OH	NHMe	3.22	—	—
[^125^I]**59e**	OH	NHMe	—	7.76	2.66
**59f**	OH	OH	71.2	—	—
**59g**	OMe	NH_2_	4.4	—	—
**59h**	OMe	NHMe	1.93	—	—
**59i**	OMe	OH	15.8	—	—
**59j**	NO_2_	NH_2_	4.6	—	—
**59k**	NO_2_	NHMe	1	—	—
**59l**	Br	NH_2_	0.67	—	—
**59m**	Br	NHMe	1.6	—	—
**59n**	OCH_2_OCH_3_	NH_2_	15.1	—	—
**59o**	CO_2_Me	NH_2_	3.34	—	—

The [^125^I]-labeled benzothiazole bithiophene **60** was synthesized by condensation of 5-bromo-2-aminobenzenethiol and 2,2'-bithiophene-5-carbaldehyde followed by installation of the radiolabel. In in vitro binding experiments, **60** displayed high affinity for both Aβ_1-40_ and Aβ_1-42_ aggregates with *K*_i_ values of 0.25 nM and 0.31 nM, respectively. In addition, it was used to clearly visualize Aβ plaques in AD brain sections and showed favorable pharmacokinetics in the brain with high uptake (3.42% ID/g at 2 min) and fast washout (0.53% ID/g at 60 min).

The [^125^I]-labeled phenyldiazenyl benzothiazole **61** was prepared via a diazo coupling reaction between **79** and **80** to give **81** followed by installation of the radiolabel (Scheme 6E) [60]. Interestingly, in in vitro binding experiments, **61** displayed higher affinity for tau aggregates (*K*_i_ = 0.48 nM) than for Aβ aggregates (*K*_i_ = 8.24 nM). Although it was used to clearly visualize NFTs in AD brain sections, further modifications will be necessary to improve the pharmacokinetics of this compound in the brain, as it showed particularly slow washout rate (2.89% ID/g at 60 min).

Three [^18^F]-labeled analogues of **56c**, [^18^F]O-FEt-PIB (**62**), [^18^F]FBTA (**63**), and [^18^F]3'-F-PIB ([^18^F]GE067, **64**) were also prepared. Compound **62** was synthesized by using the hydroxy group of **56c** to displace the tosylate of [^18^F]fluoroethyltosylate. Compound **62** had a *K*_i_ value of 0.17 nM for AD brain homogenate and was able to stain Aβ plaques in postmortem AD brain [61]. Although its biodistribution was not as good as that of **56c**, **62** still showed promise in an in vivo study using a rat model of AD [62]. Moving the [^18^F]fluoroethoxy substituent of **62** from the 6-position to the 3'-position resulted in a low binding affinity for Aβ and an inability to stain plaques in postmortem AD brain [63]. In compound **63**, the [^11^C]methylamino group of [^11^C]**56c** was replaced by a [^18^F]fluoroethylamino group, and, while this compound showed better binding affinity than **56c**, its brain pharmacokinetics were not as good [64]. Compound **64** also showed promising results in whole-body biodistribution and radiation dosimetry studies [65].

A series of fluorinated benzothiazoles **65a**–**e** was synthesized by direct substitution of the nitro group of a key synthetic intermediate **82** (prepared using synthetic steps already describe for [^11^C]**56c**) by an [^18^F] atom (Scheme 6F) [66–67]. Compounds **65a**,**b**,**d** (R = H, Me, and OMe) all showed high binding affinity for AD brain homogenates with *K*_i_ values below 10 nM, which is comparable to that of **56c** in the same assay, while **65c** (R = OH) showed slightly reduced affinity (Table 8). In addition to showing a promising ability to stain Aβ plaques in vivo, [^18^F]**65a**,**b** showed high brain uptake and rapid washout in normal mice. In fact, each of these compounds displayed better pharmacokinetics than [^11^C]**56c** in the same assay.

**Table 8:** Inhibition constants and biodistribution of radioactivity of fluorinated 2-arylbenzothiazole derivatives **65a**–**e** (values are from [66–67]).

Compound	R	human AD brain homogenates *K*_i_ (nM)	%ID/g at 2 min	%ID/g at 30 min

**65a**	H	9.0 ± 2.0	—	—
[^18^F]**65a**	H	—	3.20 ± 0.38	0.21 ± 0.03
**65b**	Me	5.7 ± 1.8	—	—
[^18^F]**65b**	Me	—	5.33 ± 0.74	0.27 ± 0.06
**65c**	OH	22.5 ± 4.5	—	—
[^18^F]**65c**	OH	—	4.70 ± 0.48	0.57 ± 0.36
**65d**	OMe	2.2 ± 0.5	—	—
[^18^F]**65d**	OMe	—	5.10 ± 0.40	0.43 ± 0.12
**65e**	CO_2_H	>4000	—	—

**Scheme 7:**
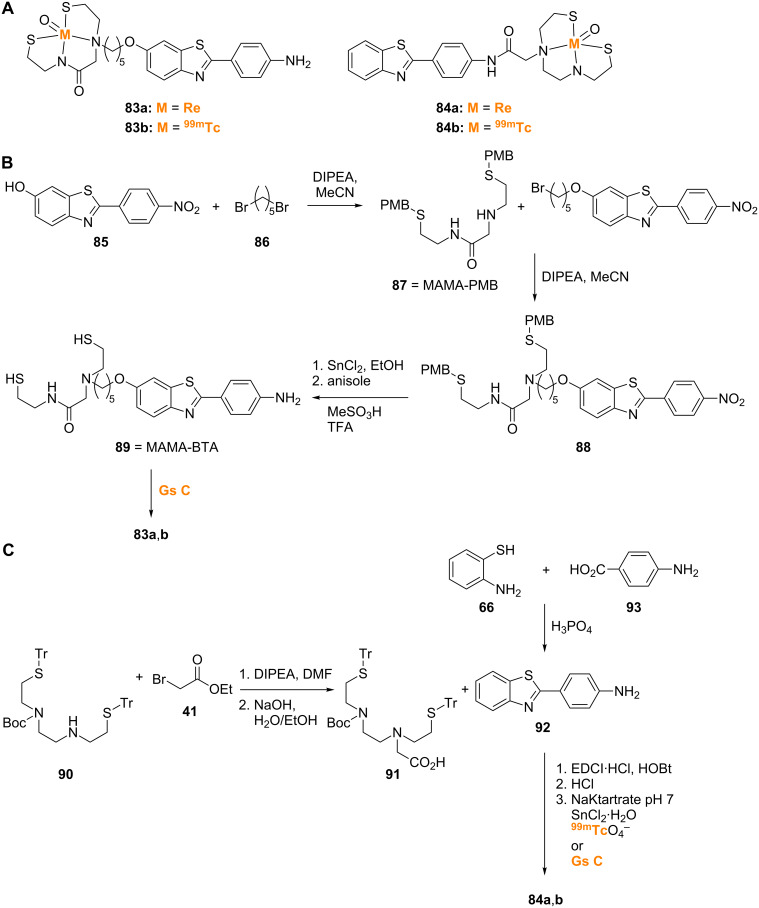
**A**. Structures of the [Re]- and [^99m^Tc]-labeled benzothiazole analogues discussed. **B**.,**C**. Synthetic schemes for the preparation of **83a**,**b** and **84a**,**b**.

Benzothiazole probes such as **83a**,**b** and **84a**,**b** labeled with [Re] and [^99m^Tc] were also synthesized (Scheme 7A) [68–69]. [Re] and [^99m^Tc]MAMA-BTA (**83a** and **83b**; MAMA = monoamine-monoamide bisthiol-BTA) were prepared by first linking 2-(4-nitrophenyl)-6-hydroxybenzothiazole (**85**) via 1,5-dibromopentane (**86**) to monoamine-monoamide bisthiol protected with *p*-methoxy benzyl (MAMA-PMB, **87**) to give **88** (Scheme 7B) [68]. Nitro reduction of **88** followed by thioether deprotection gave MAMA-BTA (**89**), which was labeled through reaction with the [Re] (used for in vitro studies) or [^99m^Tc] precursors to give the desired **83a**,**b**. [Re] and [^99m^Tc]BAT-BTA (**84a** and **84b**; note: BAT = bis(aminoethanethiol)) were prepared by addition of ethyl bromoacetate (**41**) to the unprotected amine of the *S*,*S*'-bis-trityl-*N*-Boc-1,2-ethylenedicysteamine chelating agent (**90**) followed by saponification that gave the free acid intermediate **91**, which was coupled with 2-(4-aminophenyl)-1,3-benzothiazole (**92**) (prepared from 2-aminothiophenol (**66**) and 4-aminobenzoic acid (**93**)) by using EDCI·HCl and HOBt (Scheme 7C) [69]. Deprotection followed by reaction with the [Re] or [^99m^Tc] precursors gave **84a** and **84b**. While both **83a** and **84a** showed promise as in vitro Aβ labeling agents, the [^99m^Tc] analogues **83b** and **84b** exhibited problems in pharmacokinetic studies in vivo. Compound **83b** showed sufficient initial uptake (1.34% ID/g at 2 min), but delayed washout (0.65% ID/g at 60 min) in normal mice, while **84b** was unable to cross the BBB to a sufficient degree.

#### Benzoxazoles

**Figure 3:**
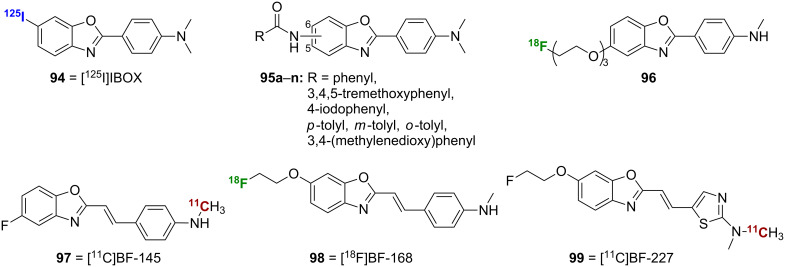
Structures of the radiolabeled benzoxazole analogues discussed.

**Scheme 8:**
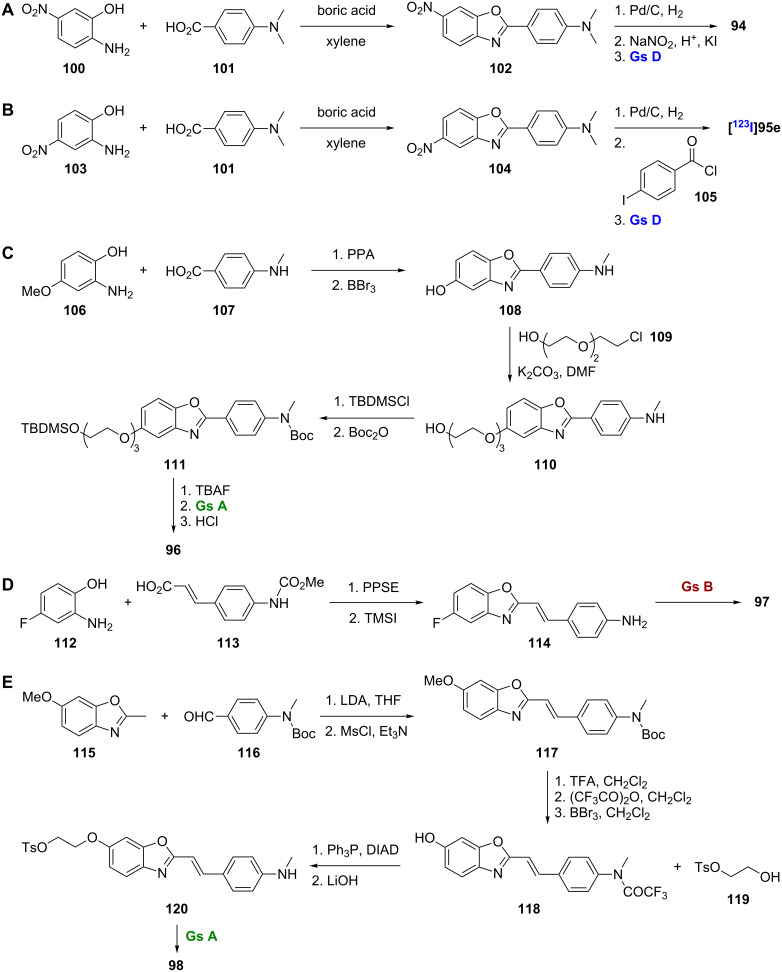
**A**.–**E**. Synthetic schemes for the preparation of **94**, [^123^I]**95e**, **96**–**98**.

Replacement of the sulfur of the benzothiazole backbone by oxygen affords the benzoxazole backbone. Compounds **94**, **95a**–**n**, and **96–99** (Figure 3) have also been successfully employed for radioimaging of Aβ plaques. The isosteric replacement of the sulfur of [^125^I]TZDM (**58a**) with an oxygen was designed to decrease molecular weight and increase lipophilicity and afforded [^125^I]IBOX (**94**) [70]. Compound **94** was prepared via boric acid catalyzed condensation of 5-nitro-2-aminophenol (**100**) and 4-dimethylaminobenzoic acid (**101**) to give the nitro intermediate **102**, which was reduced through catalytic hydrogenation to the amine (Scheme 8A). Subsequent conversion to the diazonium ion and displacement with iodide ion gave IBOX, which was radiolabeled to give **94**. Compound **94** showed similar affinity for Aβ_1-40_ aggregates when compared to **58a**, and it was able to label Aβ plaques in postmortem AD brain sections. Importantly, **94** showed superior peak brain uptake (2.08% ID/g at 30 min) and faster brain washout than **58a** in normal mice.

Expanding on this 2-arylbenzoxazole scaffold, a series of benzamide-substituted 2-arylbenzoxazoles **95a**–**n** was synthesized [71]. A representative synthesis of [^123^I]**95e** is shown (Scheme 8B). Boric acid catalyzed condensation of 4-nitro-2-aminophenol (**103**) and 4-dimethylaminobenzoic acid (**101**) gave the nitro intermediate **104**. Catalytic hydrogenation as above gave the amine intermediate, and subsequent reaction with 4-iodobenzoyl chloride (**105**) and installation of the radiolabel gave the target compound. SAR analysis of the compounds indicates that the benzamide moiety is favored at position 5 rather than 6 of the benzoxazole core in terms of binding affinity for Aβ plaques in vitro (Table 9). The best compound was **95e**, which had a *K*_i_ value of 9.3 nM, but [^123^I]**95e** was unable to cross the BBB in vivo. This disappointing result could be appointed to the excessively high lipophilicity of the compound.

**Table 9:** Inhibition constants of benzamide-substituted 2-arylbenzoxazole derivatives **95a**–**n** (values are from [71]).

Compound	R	*K*_i_ (nM)

**95a**	5-phenyl	12.0
**95b**	6-phenyl	26.0
**95c**	5-(3,4,5-trimethoxyphenyl)	109
**95d**	6-(3,4,5-trimethoxyphenyl)	628
**95e**	5-(4-iodophenyl)	9.3
**95f**	6-(4-iodophenyl)	60.1
**95g**	5-(*p*-tolyl)	13.2
**95h**	6-(*p*-tolyl)	86.0
**95i**	5-(*m*-tolyl)	13.4
**95j**	6-(*m*-tolyl)	31.5
**95k**	5-(*o*-tolyl)	18.9
**95l**	6-(*o*-tolyl)	112
**95m**	5-(3,4-(methylenedioxy)phenyl)	17.2
**95n**	6-(3,4-(methylenedioxy)phenyl)	19.7

To improve the pharmacokinetic profile of **94**, the [^18^F]-labeled analogue **96** was designed as an imaging probe [72]. Compound **96**, which contains an [^18^F] end-capped polyethylene glycol chain at position 5 of the benzoxazole core in place of the [^125^I] of **94** at position 6 to reduce lipophilicity, was prepared by polyphosphoric acid catalyzed condensation of 2-amino-4-methoxyphenol (**106**) and 4-monomethylaminobenzoic acid (**107**) to give the benzoxazole core, which was *O*-demethylated to give **108** (Scheme 8C). Subsequent coupling with 2-[2-(2-chloroethoxy)ethoxy]ethanol (**109**) gave **110**. TBDMS protection of the alcohol and Boc protection of the amine gave **111**. Finally, TBAF cleavage, installation of the radiolabel, and acid cleavage gave **96**. Compound **96** showed good affinity for Aβ_1-42_ (*K*_i_ = 9.3 nM). This compound also showed promising pharmacokinetics in normal mice, with greatly improved uptake and washout rates compared to **94**, and it successfully labeled Aβ plaques in vitro. In addition, it showed increased retention in vivo in transgenic AD mice compared to wild-type. A *N*,*N*-dimethyl derivative was also synthesized, and, while it too showed good affinity for Aβ_1-42_, its increased lipophilicity compared to the monomethyl compound gave slightly worse pharmacokinetic properties.

The [^11^C]-labeled styrylbenzoxazole [^11^C]BF-145 (**97**) and the related [^18^F]-labeled styrylbenzoxazole [^18^F]BF-168 (**98**) were prepared and studied for Aβ imaging [73–75]. The simple two-step synthesis of **97** used polyphosphoric acid trimethylsilyl ester (PPSE) catalyzed condensation of 4-fluoro-2-aminophenol (**112**) with a cinnamic acid **113** to give the benzoxazole core followed by conversion to the primary amine **114** and radiolabeling (Scheme 8D). The synthesis of **98** was more complex and began with a reaction between 2-methyl-6-methoxybenzoxazole (**115**) and 4-((*N*-Boc-*N*-methyl)amino)benzaldehyde (**116**) followed by a dehydration reaction to give **117** (Scheme 8E). Subsequent removal of the Boc group followed by installation of a trifluoroacetamide and *O*-demethylation gave the intermediate **118** used in a Mitsunobu reaction with 2-hydroxyethyl tosylate (**119**). Amine deprotection to **120** and installation of the [^18^F] label gave the target compound **98**.

Both **97** and **98** showed good affinity for Aβ_1-42_ aggregates (*K*_i_ = 4.5 nM and 6.4 nM, respectively). Interestingly, while **98** was able to selectively stain senile plaques (SPs) and NFTs in AD brain sections, **97** was only able to stain SPs. In addition, **97** and **98** showed substantial brain uptake and fast washout (4.4% and 3.9% ID/g at 2 min and 1.6% and 1.6% ID/g at 30 min, respectively) with promising in vivo imaging results in transgenic mice.

Building on the promising results of **97** and **98**, an optimized derivative, [^11^C]BF-227 (**99**), was studied for Aβ imaging. The key difference in **99** is the replacement of a phenyl ring with a thiazole ring. Compound **99** demonstrated good affinity for synthetic Aβ_1-42_ aggregates (*K*_i_ = 4.3 nM), rapid uptake (7.9% ID/g at 2 min) and clearance (0.64% ID/g at 60 min) in normal mice, the ability to selectively stain Aβ plaques in AD brain sections, and promising results in a clinical PET study in AD patients [76]. Additional studies suggest that **99** has the possibility to be useful for early detection of AD and also for predicting progression from mild cognitive impairment to AD [77–78]. Interestingly, **99** has also shown promise for diseases other than AD. It has been suggested that **99** may provide a means of diagnosis and disease monitoring in transmissible spongiform encephalopathies [79] and may be useful for monitoring α-synuclein deposits in conditions such as multiple system atrophy and Parkinson’s disease [80]. A version of **99** labeled with [^18^F] rather than [^11^C] has also been proposed for use in Parkinson’s disease [81].

#### Benzofurans

**Figure 4:**
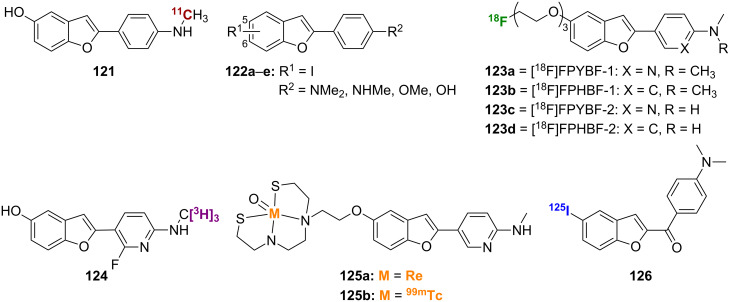
Structures of the radiolabeled benzofuran analogues discussed.

**Scheme 9:**
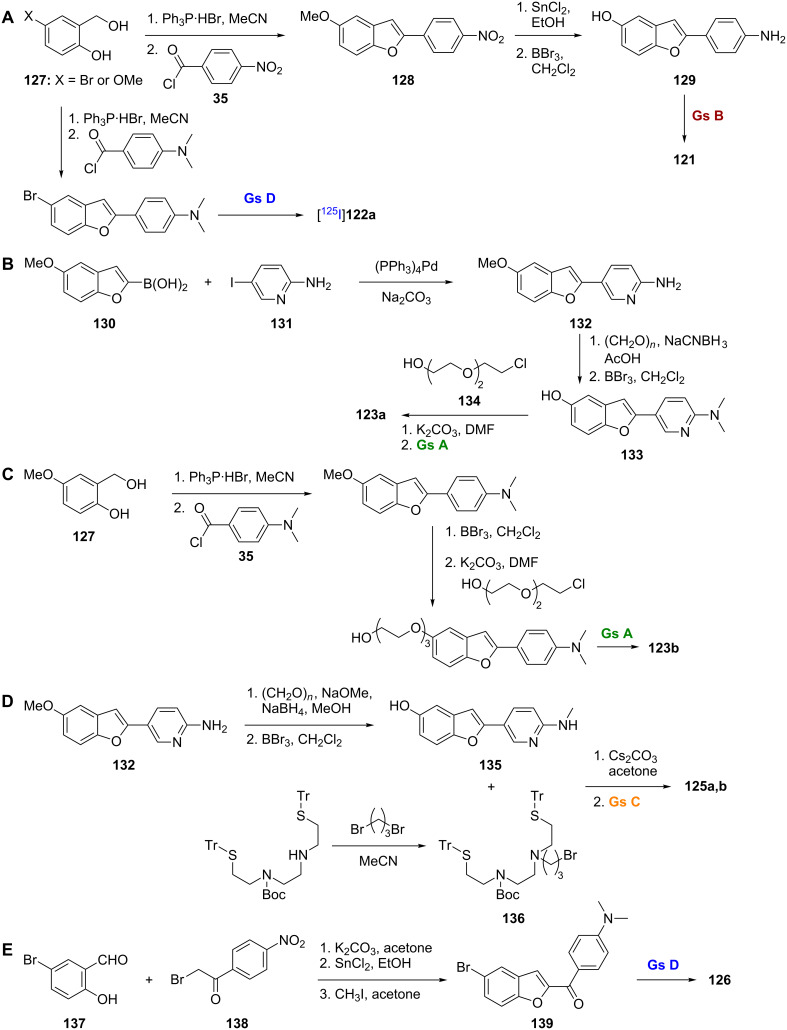
**A**.–**E**. Synthetic schemes for the preparation of **121**, [^125^I]**122a**, **123a**,**b**, **125a**,**b**, and **126**.

Replacement of the nitrogen of the benzoxazole backbone with carbon affords the benzofuran backbone of compounds **121**–**126** (Figure 4), which has also been successfully employed for radioimaging of Aβ plaques. The [^11^C]-labeled benzofuran **121** was prepared via Wittig reaction between the triphenylphosphonium salt of 2-hydroxy-5-methoxybenzyl alcohol (**127**) and 4-nitrobenzoyl chloride (**35**) to give **128** followed by nitro reduction and *O*-demethylation to give **129** and radiolabeling (Scheme 9A). Using AD brain gray matter homogenates, compound **121** showed good binding affinity for Aβ plaques (*K*_i_ = 0.7 nM) and was able to stain both SPs and NFTs in vitro. In normal mice, this compound showed rapid uptake (4.8% ID/g at 2 min) and fast washout (0.2% ID/g at 60 min). In vivo plaque labeling in APP transgenic mice was also successful [82].

Using similar chemistry as described above, a series of iodinated 2-arylbenzofurans **122a**–**e** was prepared and studied [83]. A representative synthesis of [^125^I]**122a**, which uses a similar Wittig as in the synthesis of **121**, is shown in Scheme 9A. It was found that the iodo substituent could be varied between the 5- and 6-positions, and the *N*,*N*-dimethylamino substituent could be changed to a secondary methylamino or hydroxy moiety with little effect on binding affinity for synthetic Aβ_1-40_, as all compounds had a *K*_i_ ≤ 8 nM (Table 10). While the [^125^I]-labeled benzofurans in this series showed good brain uptake in normal mice, their washout was rather slow indicating nonspecific binding in vivo.

**Table 10:** Inhibition constants and biodistribution of the radioactivity of iodinated 2-arylbenzofuran derivatives **122a**–**e** (values are from [83]).

Compound	R^1^	R^2^	Aβ_1-40_* K*_i_ (nM)	%ID at 2 min	%ID at 60 min

**122a**	5-iodo	NMe_2_	7.7 ± 1.2	—	—
[^125^I]**122a**	5-iodo	NMe_2_	—	0.51 ± 0.05	1.08 ± 0.15
**122b**	5-iodo	NHMe	1.1 ± 0.2	—	—
[^125^I]**122b**	5-iodo	NHMe	—	0.78 ± 0.06	1.20 ± 0.34
**122c**	5-iodo	OMe	4.2 ± 0.8	—	—
**122d**	5-iodo	OH	6.5 ± 0.2	—	—
[^125^I]**122d**	5-iodo	OH	—	1.40 ± 0.04	1.51 ± 0.20
**122e**	6-iodo	NMe_2_	0.4 ± 0.1	—	—
[^125^I]**122e**	6-iodo	NMe_2_	—	0.48 ± 0.07	1.00 ± 0.22

Several [^18^F]-labeled benzofurans have been employed with success for Aβ imaging. [^18^F]FPYBF-1 (**123a**), which has a *N*,*N*-dimethyl-2-aminopyridine group attached to the benzofuran core, was synthesized via Suzuki coupling between 5-methoxybenzofuran-2-boronic acid (**130**) and 2-amino-5-iodopyridine (**131**) to give **132**, which was followed by reductive amination and *O*-demethylation to give **133** (Scheme 9B). Finally, reaction with 2-[2-(2-chloroethoxy)ethoxy]ethanol (**134**) followed by radiolabeling gave **123a**. This compound showed high affinity for Aβ_1-42_ aggregates (*K*_i_ = 0.9 nM), the ability to label plaques in postmortem AD brains, and suitable pharmacokinetic properties in normal mice (5.16% ID/g at 2 min and 2.44% ID/g at 60 min). In addition, it showed good in vivo binding to plaques in transgenic mice [84]. A closely related compound, [^18^F]FPHBF-1 (**123b**), which has a *N*,*N*-dimethylaniline group in place of the *N,N*-dimethylaminopyridine group, was prepared using a Wittig reaction between the triphenylphosphonium salt of 2-hydroxy-5-methoxybenzyl alcohol (**127**) and 4-dimethylaminobenzoyl chloride (**35**), followed by *O*-demethylation and installation of the [^18^F]-labeled linker (Scheme 9C). Like **123a**, compound **123b** showed good affinity for Aβ aggregates in vitro and in vivo. However, its slow washout from the brain, which can be attributed to its increased lipophilicity compared to **123a**, indicated that additional refinements will be needed [85].

Derivatives of **123a** and **123b** were also prepared [86]. These compounds, [^18^F]FPYBF-2 (**123c**) and [^18^F]FPHBF-2 (**123d**), have a secondary methylamino group in place of the dimethylamino group. Introduction of the secondary amine served to reduce lipophilicity. In addition, as the secondary amines are less rapidly metabolized than the tertiary amines, they may help improve the stability of these compounds in vivo. The synthesis of these derivatives used methodology similar to that already described for **123a** and **123b**. One key difference, however, was the need for orthogonal TBS and Boc protection/deprotection to prevent the secondary amine from reacting with the MsCl used to introduce the radiolabel. Both **123c** and **123d** showed good affinity for Aβ_1-42_ aggregates (*K*_i_ = 2.41 nM and 3.85 nM, respectively) as well as the ability to label plaques in transgenic mice. Also, both **123c** and **123d** showed high uptake and rapid washout with improved pharmacokinetic properties when compared to **123a** and **123b**.

The [^3^H]-labeled AZD4694 (**124**) also showed promise for Aβ imaging [87]. With good affinity for β-amyloid fibrils in vitro (*K*_d_ = 2.3 nM), this compound was able to label plaques in human AD brain sections with little nonspecific binding. In addition, the good pharmacokinetic profile of **124** warrants further investigation in vivo.

[Re] and [^99m^Tc]-labeled benzofurans, BAT-Bp-2 (**125a**,**b**), were synthesized from **132** by reductive monoamination and *O*-demethylation to give **135** (Scheme 9D) [88]. Subsequent reaction with the protected chelation ligand TRT-Boc-BAT-Br (**136**) and labeling through reaction with the rhenium (used for in vitro studies) and technetium precursors gave compounds **125a** and **125b**, respectively. Compound **125b** showed decent affinity for Aβ_1-42_ aggregates (*K*_i_ = 32.8 nM) in vitro, although, by comparison to other benzofuran probes of similar structure, it was clear that introduction of the BAT chelator decreased binding affinity. In contrast to other [^99m^Tc]-labeled Aβ probes, **125b** showed decent brain uptake and washout rates in normal mice (1.80% ID/g at 2 min and 0.79% ID/g at 60 min). In addition, it was able to label Aβ plaques in vivo in transgenic mice, a first for a [^99m^Tc]-labeled Aβ probe.

The [^125^I]-labeled probe **126** contains the benzofuran core, but could also be classified as a chalcone, specifically a chalcone in which the conformation around the double bond is fixed [89]. Compound **126** was synthesized by using a Rap–Stoermer condensation between the bromo-substituted salicylaldehyde (**137**) and α-brominated 4-nitroacetophenone (**138**) to form the benzofuran core (Scheme 9E). Nitro reduction followed by methylation gave **139**, and radiolabeling gave **126**. This compound showed good affinity for Aβ_1-42_ aggregates (*K*_i_ = 6.6 nM). Secondary methylamino and primary amino derivatives showed decreased binding affinity and poorer labeling of plaques in brain sections from transgenic mice. While the pharmacokinetics of this compound in normal mice were promising (3.53% ID/g at 2 min and 0.87% ID/g at 60 min), they were not as good as those previously reported for [^125^I]-labeled *N,N*-dimethylamino chalcones and aurones.

#### Imidazopyridines

**Scheme 10:**
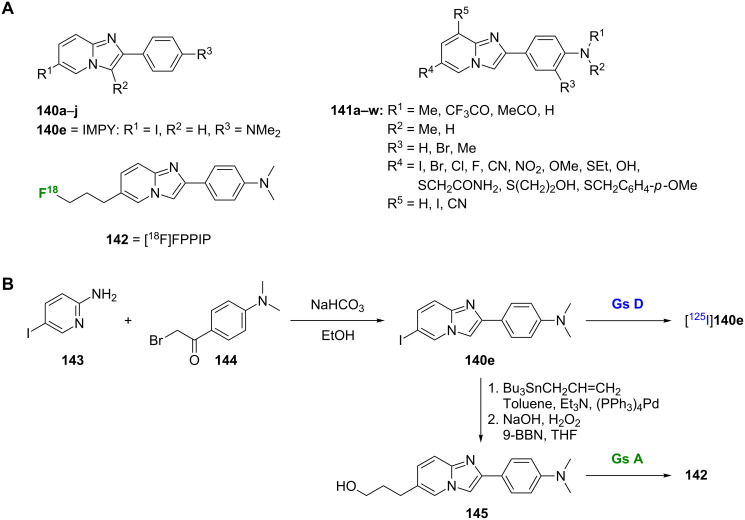
**A**. Structures of the radiolabeled imidazopyridine analogues discussed. **B**. Synthetic scheme for the preparation of [^125^I]**140e** and **142**.

The imidazopyridine core has also been used in developing novel Aβ imaging agents such as **140**–**142** (Scheme 10A). Initial SAR studies were based on derivatives **140a**–**j**. One of the most successful imidazopyridines studied to date has been [^125^I]IMPY ([^125^I]**140e**). Representative of this scaffold, the synthesis of [^125^I]**140e** used a fusion reaction between 2-amino-5-iodopyridine (**143**) and an α-bromoacetophenone **144** to form **140e**, which was then radiolabeled (Scheme 10B) [90]. This preparation has since been improved by Kung et al. who, through the use of a reverse-phase C4 minicolumn with stepwise washing and elution, have simplified the purification process by eliminating the need for HPLC purification [91]. While **140e** showed good affinity for Aβ_1-40_ aggregates (*K*_i_ = 15.0 nM), SAR analysis demonstrated that, in general, other modifications to the scaffold were not well tolerated and reduced the binding affinity (Table 11). An exception was the replacement of the 6-iodo substituent with a bromine, as **140f** showed similar affinity to the parent compound [90].

**Table 11:** Inhibition constants and biodistribution of radioactivity of 2-arylimidazopyridine derivatives **140a**–**j** (values are from [90]).

Compound	R^1^	R^2^	R^3^	Aβ_1-40_* K*_i_ (nM)	%ID at 2 min	%ID at 60 min

**140a**	H	H	NHMe	>1000	—	—
**140b**	H	H	NMe_2_	>2000	—	—
**140c**	Me	H	NHMe	>2000	—	—
**140d**	Me	H	NMe_2_	242 ± 20	—	—
**140e**	I	H	NMe_2_	15 ± 5	—	—
[^125^I]**140e**	^125^I	H	NMe_2_	—	2.88 ± 0.25	0.21 ± 0.03
**140f**	Br	H	NMe_2_	10.3 ± 1.2	—	—
**140g**	Me	H	Br	638 ± 30	—	—
**140h**	NMe_2_	H	Br	339 ± 40	—	—
**140i**	H	I	NMe_2_	>2000	—	—
**140j**	I	I	NMe_2_	>2000	—	—

In addition to its good binding affinity for Aβ, [^125^I]**140e** showed other promising properties for use as an imaging probe. For example, it selectively labeled plaques in postmortem AD brain sections and showed plaque labeling with low background activity in a transgenic mouse model. The pharmacokinetics of [^125^I]**140e** were also promising. It showed high uptake (2.9% ID/g at 2 min) and fast washout (0.2% ID/g at 60 min) in normal mice. These kinetic properties represented improvements over both **58a** and **94** [92]. Safety, biodistribution, and dosimetry studies of [^123^I]IMPY, the [^123^I]-labeled counterpart of **140e**, have indicated it may be a safe radiotracer with appropriate pharmacokinetics for use in AD patients [93].

More in depth SAR studies of the imidazopyridine scaffold have been conducted by synthesizing the series of analogues **141a**–**w** [94]. The effect of different substituents on binding affinity (*K*_i_) for human Aβ plaques was examined (Table 12). In general, it was found that the *N*,*N*-dimethylamino analogues (R^1^ = R^2^ = Me) had higher binding affinity for human Aβ plaques than did the secondary methylamino analogues (R^1^ = H, R^2^ = Me). Little tolerance for substitution at both R^3^ and R^5^ was seen, as the most potent compounds almost always had a hydrogen atom at these positions. One exception was observed with the secondary methylamino analogue when R^3^ = Br, as **141n** showed high affinity. For R^4^ it was seen that polarizable or electron-withdrawing substituents showed higher affinity than strongly electron-donating substituents. In addition, it was observed that bulky, hydrophobic thioether substituents (such as R^4^ = SCH_2_C_6_H_4_-*p*-OMe) were well tolerated at this position. This finding was of particular interest as it provided a possible means of generating new PET ligands via [^11^C]- or [^18^F]-labeling through *S*-alkylation.

**Table 12:** Inhibition constants of 2-arylimidazopyridine derivatives **141a**–**w** (values are from [94]).

Compound	R^1^	R^2^	R^3^	R^4^	R^5^	human AD brain homogenates *K*_i_ (nM)

**140e**	Me	Me	H	I	H	8.9 ± 0.7
**141a**	Me	Me	H	Br	H	5.9 ± 0.4
**141b**	Me	Me	H	Cl	H	24.2 ± 5.6
**141c**	Me	Me	H	F	H	13.0 ± 1.6
**141d**	Me	Me	H	CN	H	8.2 ± 1.0
**141e**	Me	Me	H	NO_2_	H	7.6 ± 0.7
**141f**	Me	Me	H	OMe	H	38.5 ± 5.0
**141g**	Me	Me	H	SEt	H	8.3 ± 0.5
**141h**	Me	Me	H	Br	I	183 ± 61
**141i**	Me	Me	H	Br	CN	>180
**141j**	Me	Me	H	OH	H	177 ± 31
**141k**	CF_3_CO	Me	Br	Br	H	>1000
**141l**	CF_3_CO	H	Me	Br	H	>1000
**141m**	MeCO	Me	Me	Br	H	>1000
**141n**	H	Me	Br	Br	H	7.4 ± 0.6
**141o**	H	H	Me	Br	H	658 ± 47
**141p**	H	Me	Me	Br	H	>1000
**141q**	H	Me	H	SCH_2_CONH_2_	H	1840 ± 497
**141r**	H	Me	H	S(CH_2_)_2_OH	H	645 ± 75
**141s**	Me	Me	H	SCH_2_CONH_2_	H	391 ± 76
**141t**	Me	Me	H	SCH_2_C_6_H_4_-*p*-OMe	H	8.3 ± 1.8
**141u**	Me	Me	H	S(CH_2_)_2_OH	H	88 ± 6
**141v**	H	Me	Me	SCH_2_CONH_2_	H	>1000
**141w**	H	Me	Me	S(CH_2_)_2_OH	H	>1000

The [^18^F]-labeled imidazopyridine, [^18^F]FPPIP (**142**), was prepared starting from **140e**. A palladium-catalyzed coupling with tributyl(vinyl)tin to give an alkene intermediate was followed by hydroboration-oxidation to give the hydroxypropyl intermediate **145**, which was radiolabeled to give **142** (Scheme 10B). This compound showed good binding affinity for Aβ (*K*_i_ = 48.3 nM) in using human AD cortical tissues, as well as specific labeling of Aβ plaques in postmortem AD brain. This, coupled with favorable pharmacokinetics observed in a normal rhesus monkey, made **142** a promising compound [95]. However, another [^18^F]-labeled imidazopyridine, [^18^F]FPM-IMPY, has shown less promising results. This compound, in which one of the *N*-methyl groups of IMPY was replaced with a [^18^F]fluoropropyl moiety, showed lower binding affinity than IMPY and poor pharmacokinetics [96].

#### Benzimidazoles

**Scheme 11:**
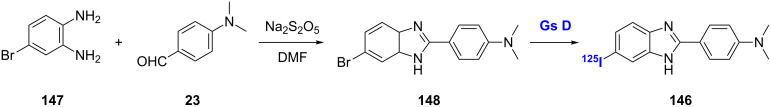
Synthetic scheme for the preparation of the benzimidazole **146**.

The benzimidazole scaffold is highly similar to the imidazopyridine scaffold, but the benzimidazole ring has reduced lipophilicity when compared to imidazopyridines. This has the potential to reduce nonspecific binding and enhance signal-to-noise ratio. The [^125^I]-labeled benzimidazole analogue of **140e**, compound **146**, was prepared through cyclization of 4-bromobenzene-1,2-diamine (**147**) and *p*-dimethylaminobenzaldehyde (**23**) to give **148** followed by installation of the radiolabel (Scheme 11) [97]. Compound **146** showed good binding affinity for Aβ_1-42_ aggregates (*K*_i_ = 9.8 nM), as well as high uptake and rapid clearance in normal mice (4.14% ID/g at 2 min and 0.15% ID/g at 60 min). In vitro labeling of Aβ plaques in AD brain sections showed a strong signal with low background, and in vivo plaque labeling in transgenic mice was also successful. However, this scaffold is lacking in detailed SAR analysis compared to the imidazopyridine scaffold.

### Quinoline and naphthalene analogues

#### Quinolines

**Figure 5:**
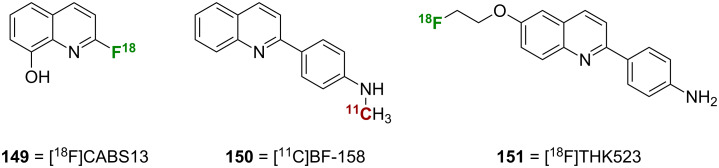
Structures of the quinolines discussed.

Investigation of the quinoline scaffold for imaging in AD has yielded some interesting results, despite there only being a few examples in the literature. The [^18^F]-labeled 2-fluoroquinolin-8-ol [^18^F]CABS13 (**149**) has recently been reported (Figure 5) [98]. The straightforward synthesis of this compound began with benzyl protection of 2-chloroquinolin-8-ol followed by installation of the [^18^F]-label and Pd-catalyzed hydrogenolysis to give the target compound. Compound **149** potently bound to Aβ-Zn aggregates (*K*_d_ = 1.5 nM) and showed rapid uptake and washout in normal mice (10% ID/g at 2 min and 1.1% ID/g at 30 min). Also, delayed washout of **149** was observed in APP/Ps1 transgenic mice, which was indicative of non-specific binding to Aβ plaques. However, two other quinoline probes, [^11^C]BF-158 (**150**) and [^18^F]THK523 (**151**), had high affinity for tau pathology as opposed to Aβ. Compound **150** showed good uptake and washout in normal mice (11.3% ID/g at 2 min and 2.1% ID/g at 60 min), and was able to label NFTs in postmortem AD brain section while only faintly staining plaques [99]. Compound **151** showed high affinity (*K*_d_ = 1.7 nM) and selectivity for recombinant tau fibrils in vitro, and, with favorable pharmacokinetics, it was able to highlight tau pathology in vivo in transgenic mice [100].

#### Naphthalenes

**Scheme 12:**
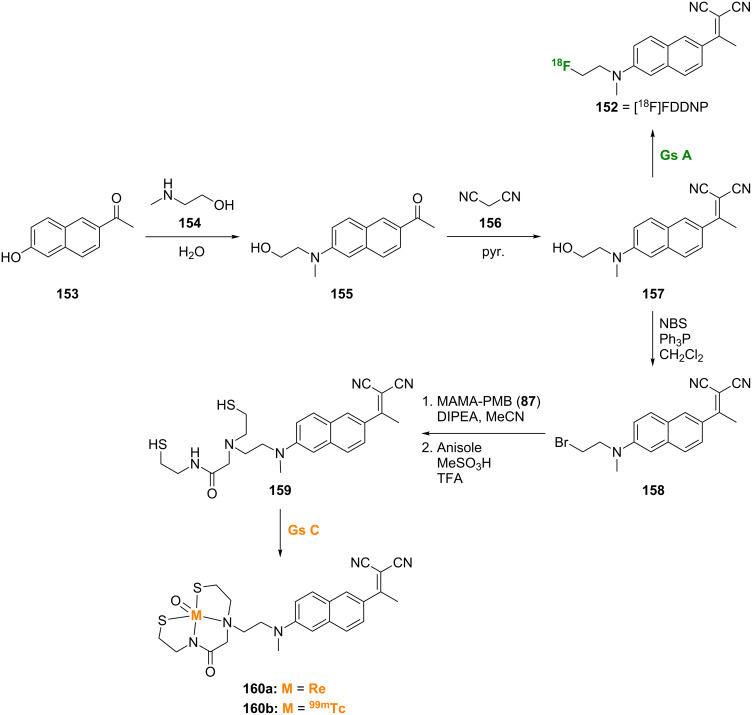
Synthetic scheme for the preparation of the naphthalene analogues **152** and **160a**,**b**.

Replacement of the cyclic nitrogen in the quinoline scaffold described in the previous section affords the naphthalene scaffold. This scaffold has shown promising results for Aβ imaging, particularly [^18^F]FDDNP (**152**), although this scaffold is also represented by only a few examples in the literature. Compound **152** was prepared starting from 1-(6-hydroxy-2-naphthyl)-1-ethanone (**153**) via a Bucherer reaction with 2-(methylamino)ethanol (**154**) followed by Knoevenagel reaction of **155** with malononitrile (**156**) and [^18^F] labeling of **157** (Scheme 12) [101–102]. Compound **152** bound to synthetic Aβ_1-40_ fibrils with high affinity (*K*_d_ = 0.12 nM) and crossed the BBB [103]. In addition, PET imaging studies using **152** demonstrated the ability of this compound to determine the localization and load of both SPs and NFTs in living AD patients [104], as well as the ability to differentiate between patients with no cognitive impairment, mild cognitive impairment, and AD [105].

[Re]- and [^99m^Tc]-labeled derivatives of **152** have also been prepared by bromination of **157** with NBS to give **158** followed by conjugation with MAMA-PMB (**87**) and deprotection with acid to give **159**. Reaction with the technetium or rhenium precursors gave the target derivatives **160a**,**b** (Scheme 12) [106]. In vitro binding studies with the [Re]-labeled compound **160a** showed a 14-fold decrease in binding affinity for Aβ_1-42_ aggregates compared to **152**. In addition, the [^99m^Tc]-labeled compound **160b** showed very low brain uptake in normal mice indicating the need for additional refinements of this compound.

### Combination of known scaffolds

**Scheme 13:**
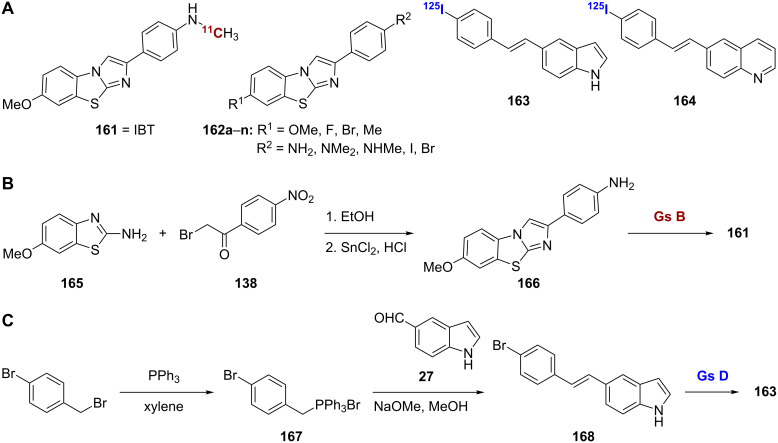
**A**. Structures of the radiolabeled analogues resulting from the combination of various scaffolds. **B**.,**C**. Synthetic schemes for the preparation of **161** and **163**.

With the success of the benzothiazole and imidazopyridine scaffolds for Aβ imaging, it was logical to suspect that combination of the two scaffolds into a single molecule could also provide a good imaging agent. Examples of this combination scaffold can be seen in compounds **161**–**164** (Scheme 13A). IBT (**161**) was prepared by direct coupling of 6-methoxybenzo[*d*]thiazol-2-amine (**165**) and the nitro substituted α-bromoacetophenone **138** to give **166** followed by installation of the radiolabel (Scheme 13B) [107]. Compound **161** showed good affinity for both Aβ_1-40_ and Aβ_1-42_ (*K*_i_ = 3.5 nM and 5.8 nM, respectively) and was comparable to compound **56c** in the same assay. The pharmacokinetics of this compound were also similar to those of [^11^C]**56c**. In vivo specific plaque labeling by compound **161** was confirmed through studies in APP/Ps1 transgenic mice. Derivatives of this combination scaffold **162a**–**n** were also investigated (Table 13) [108]. Of note was derivative **162i** in which the secondary methylamino group of **161** has been replaced with iodine. This derivative showed high affinity for Aβ_1-40_ (*K*_d_ = 10.9 nM), and the iodo substituent could readily be radiolabeled. However, the high lipophilicity of this compound may lead to nonspecific plaque labeling in vivo.

**Table 13:** Inhibition constants of 2-arylimidazobenzothiazole derivatives **162a**–**n** (values are from [108]).

Compound	R^1^	R^2^	Aβ_1-40_* K*_i_ (nM)

**162a**	OMe	NH_2_	29.8 ± 2.1
**162b**	OMe	NMe_2_	58.6 ± 4.7
**162c**	F	NH_2_	133 ± 21
**162d**	F	NHMe	38.1 ± 2.6
**162e**	F	NMe_2_	42.9 ± 5.7
**162f**	Br	NH_2_	28.8 ± 1.2
**162g**	Br	NHMe	34.5 ± 3.5
**162h**	Br	NMe_2_	43.4 ± 5.7
**162i**	OMe	I	10.9 ± 0.18
**162j**	F	I	41.9 ± 5.2
**162k**	Br	I	21.1 ± 0.9
**162l**	Me	I	17.7 ± 1.9
**162m**	OMe	Br	9.40 ± 0.07
**162n**	Me	Br	26.0 ± 0.9

The [^125^I]-labeled styrylindole **163** and styrylquinoline **164** scaffolds synthesized by Yang et al. can be thought of as stilbene combination scaffolds [109]. The synthesis of the [^125^I]-labeled styrylindole **163** used a Wittig reaction between the triphenyl phosphonium ylide **167** and 1*H*-indole-5-carbaldehyde (**27**) to give **168** followed by radiolabeling (Scheme 13C). The [^125^I]-labeled styrylquinoline **164** was prepared by using an identical synthesis with substitution of the indole by quinoline-6-carbaldehyde. Both **163** and **164** showed good affinity for Aβ_1-40_ aggregates (*K*_i_ = 4.1 nM and 8.6 nM, respectively). Compound **163** was able to stain Aβ plaques in in vitro brain sections from APP/Ps1 transgenic mice and showed high uptake and rapid clearance in normal mice (4.27% ID/g at 2 min and 0.28% ID/g at 60 min). However, compound **164** showed relatively low brain uptake and slow washout by comparison.

### Others

**Scheme 14:**
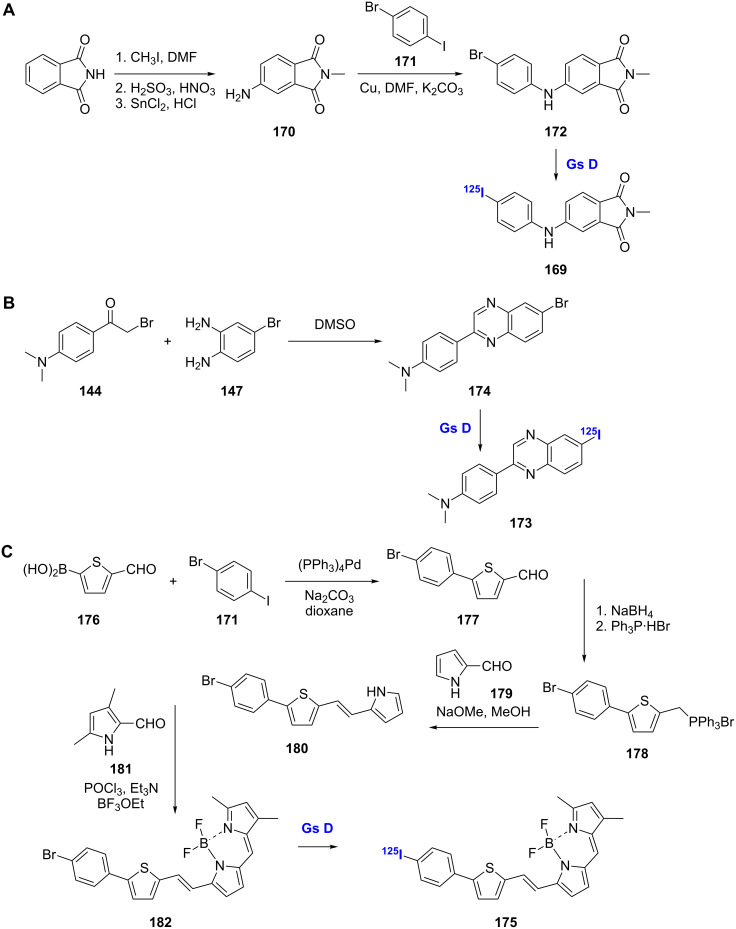
**A**.–**C**. Synthetic schemes for the preparation of radiolabeled probes with unique scaffolds.

Several other less common scaffolds have been evaluated as Aβ-imaging agents. The [^125^I]-labeled *N*-methyl-4-anilinophthalimide derivative **169** was prepared and evaluated as a potential probe for Aβ plaques (Scheme 14A) [110]. This compound was generated via a Cu powder-catalyzed coupling reaction between *N*-methyl-4-aminophthalimide (**170**) and 1-bromo-4-iodobenzene (**171**) to give **172**, which was then radiolabeled. Compound **169** showed high binding affinity to AD brain homogenates (*K*_d_ = 0.21 nM) as well as excellent brain uptake (5.16% ID/g at 2 min) and fast washout (0.59% ID/g at 60 min). SAR studies with other *N*-methyl-4-anilinophthalimide derivatives demonstrated that a hydrophobic substituent at the 4-position of the aniline ring is important for the binding affinity of this family of compounds.

The [^125^I]-labeled quinoxaline derivative **173** was also synthesized and evaluated for in vivo imaging of Aβ plaques (Scheme 14B) [111]. The quinoxaline backbone of this compound was prepared from the reaction of α-bromoacetophenone **144** and 4-bromobenzene-1,2-diamine (**147**) in DMSO in a one-pot tandem oxide condensation procedure. This reaction gave the desired 2-aryl-6-substituted quinoxaline **174** as the major product and the isomeric 2-aryl-7-substituted quinoxaline (not shown) as a minor product. The radioiodinated probe was prepared from **174**. Compound **173** showed excellent affinity for Aβ_1-42_ aggregates in vitro (*K*_i_ = 4.1 nM). In addition to being able to specifically label plaques in brain sections from AD patients, **173** readily crossed the BBB showing high uptake into the brain (6.03% ID/g at 2 min). However, with moderate washout (1.12% ID/g at 120 min), additional refinements will be needed to improve the pharmacokinetics of these molecules.

The boron [^125^I]-labeled dipyrro-methane (BODIPY) analogue **175** was prepared to serve as a dual functional SPECT/fluorescence probe for imaging Aβ (Scheme 14C) [112]. Compound **175** was synthesized through Suzuki coupling of the starting boronic acid **176** with 1-bromo-4-iodobenzene (**171**). Aldehyde reduction of **177** followed by reaction with triphenylphosphine gave the Wittig reagent **178** for reaction with 2-formylpyrrole (**179**). The Wittig product **180** was condensed with 3,5-dimethylpyrrole-2-carboxaldehyde (**181**) to form the BODIPY backbone **182**. Subsequent installation of the radiolabel gave **175**. Although **175** showed decent affinity for Aβ_1-42_ aggregates (*K*_i_ = 108 nM) and the ability to label plaques in brain sections from transgenic mice, its in vivo use was limited by extremely low brain uptake, which could be attributed to rapid trapping of the compound in the liver.

### Fluorescence probes

Although PET is currently the most promising approach for Aβ plaque detection, this technique has two main limitations: (i) the short half-life of positron-emitting nuclei (*t*_1/2_ = 20 min for [^11^C] and 110 min for [^18^F]) and (ii) the narrow availability of this technology that requires a local cyclotron for generating short-lived positron-emitting radionuclides and a synthetic unit to produce radiolabeled agents [113]. Other imaging technologies have been investigated to overcome these problems. Different fluorescence techniques have been reported [114–116]; however, the near-infrared fluorescence (NIRF) imaging technique is the only one that has an in vivo application. Since normal biological tissues reveal limited photon absorbance in the near-infrared region, NIRF seems to be the second most promising Aβ deposits tracer tool [117]. In the following sections, we will briefly cover the different scaffolds that have been explored as NIRF ligands using in vivo models.

#### Oxazines

**Scheme 15:**
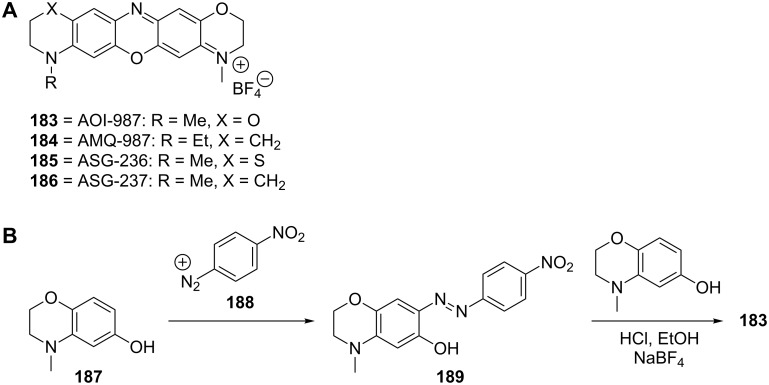
**A**. Structures of the oxazine-derived fluorescence probes discussed. **B**. Synthetic scheme for the preparation of the oxazine analogue **183**.

The oxazine dyes **183**–**186** were investigated as Aβ aggregate target-specific probes in the NIRF imaging technique (Scheme 15A) [113]. The preparation of **183** was accomplished through reaction of 4-methyl-3,4-dihydro-2*H*-benzo[*b*][1,4]oxazin-6-ol (**187**) with *p*-nitrobenzenediazonium ion (**188**) to give the key azo intermediate **189**, which afforded the desired oxazine dye **183** upon further reaction with 4-methyl-3,4-dihydro-2*H*-benzo[*b*][1,4]oxazin-6-ol (**187**) (Scheme 15B). Compound **183** proved to give a higher fluorescence intensity than other derivatives such as **184**–**186** [113]. Using APP23 transgenic mice and compound **183**, Aβ plaques could be traced quantitatively [113].

#### Thiobarbitals

**Figure 6:**
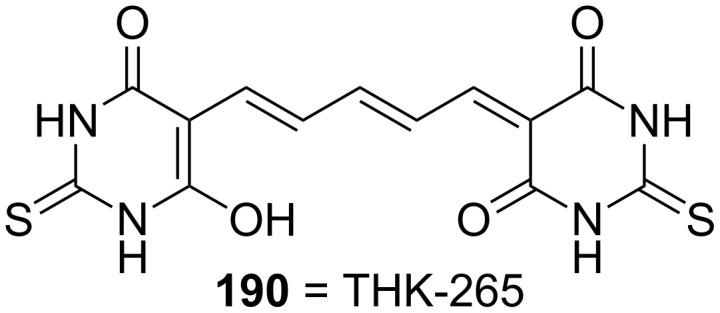
Structure of THK-265 (**190**).

The thiopental dimer THK-265 (**190**) (maximal emission wavelength >650 nm) was discovered as a good NIRF imaging ligand by screening a large pool of dye candidates (Figure 6) [117]. Compound **190** displayed high binding affinity towards Aβ aggregates (*K*_d_ = 97 nM) [117]. Its usefulness in AD diagnosis was confirmed in an animal model as it provided good discrimination between amyloid deposits in the brain and other normal tissues [117]. Compound **190** was also used in a quantitative correlation of different Aβ aggregation levels with NIRF signals [118].

#### Quinoxalines

**Scheme 16:**
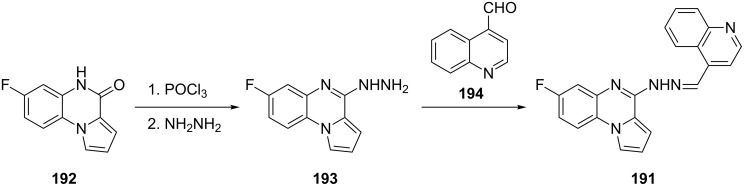
Synthetic scheme for the preparation of quinoxaline analogue **191**.

The use of the radiolabeled quinoxalines for imaging Aβ was discussed in the section “Others” of this review. A quinoxaline derivative, compound **191**, was also synthesized and explored for fluorescence imaging (Scheme 16) [119]. The synthesis of **191** began with conversion of the starting lactam **192** to the corresponding chloride by using phosphorus oxychloride followed by reaction with hydrazine. Condensation of the resulting hydrazino-derivative **193** with quinoline-4-carboxaldehyde (**194**) gave **191**. Although **191** has not been tested in vivo yet, this compound warrants further investigation as it has shown the ability to selectively stain amyloid structures in brain sections of transgenic mice, as well as the ability to cross the BBB. In addition, the 7-fluoro substituent of compound **191** could potentially be radiolabeled for in vivo application.

## Conclusion

In summary, this review covered the main scaffolds used for radioimaging of Aβ plaques, one of the major pathological hallmarks of AD. Highlighted were important synthetic steps for scaffold formation and introduction of radiolabels, SAR findings where appropriate, and binding affinities and brain kinetics of each scaffold. The synthesis of each scaffold presented was fairly straightforward using well-established reactions, and synthetic complexity will likely not impede future development of Aβ chemical probes.

Most of the scaffolds present compounds with good binding affinity for Aβ in vitro. SARs tended to vary between scaffolds so it is impossible, without extensive computational work, to declare certain functionalities necessary for this class of chemical probes. However, the dimethylamine structural feature appears in a number of the compounds with high binding affinity, and it is likely that this functionality is important for Aβ interaction. Representative examples of this can be seen in examining SAR trends for chalcones **18a**–**l**, benzothiazoles **56c**–**t**, or imidazopyridines **141a**–**w** among others. With regards to pharmacokinetics in vivo, results varied between scaffolds and radiolabels. In general, [^99m^Tc]-labeled compounds showed poor pharmacokinetic profiles with only **125b** being able to label plaques in animal studies. A balance in lipophilicity appeared to be particularly important in terms of pharmacokinetics, as imaging probes need to be lipophilic enough to easily penetrate the BBB, but not too lipophilic to avoid nonspecific binding in the brain.

Many of the molecules described in this review showed very promising results for the in vivo imaging of Aβ plaques in humans. For example, stilbenes **46a** and **46b**, benzothiazole [^11^C]**56c**, and naphthalene **152** have been studied in clinical trials with favorable results. The half-life of the radiolabel and overall lipophilicity will continue to be two of the biggest factors for the clinical success of these probes. Future development and testing of these molecules will be of critical importance as the development of Aβ imaging probes will provide an effective means of monitoring new treatments for AD. While not the focus of this review, it should be noted that the current treatments for AD only treat cognitive symptoms and have little to no effect on slowing or reversing the progression of the disease. Current research efforts aimed at developing molecules that target Aβ plaques, specifically inhibition of plaque formation and disaggregation of already formed plaques, could lead to new therapeutics capable of reversing the progression of AD. Probes such as those described herein will play an important role in evaluating the effectiveness of such drugs. Additionally, the development of Aβ imaging probes will likely lead to better and earlier diagnosis of AD, which in turn will allow future treatments to be more effective.
